# Calcium-Dependent Protein Kinase Family Genes Involved in Ethylene-Induced Natural Rubber Production in Different *Hevea brasiliensis* Cultivars

**DOI:** 10.3390/ijms19040947

**Published:** 2018-03-22

**Authors:** Liping Zhu, Xiang Jin, Quanliang Xie, Qi Yao, Xuchu Wang, Hongbin Li

**Affiliations:** 1 College of Life Sciences, Key Laboratory of Xinjiang Phytomedicine Resource Utilization of Ministry of Education, Shihezi University, Shihezi 832003, China; zhuliping0903@163.com (L.Z.); jinxiang@itbb.org.cn (X.J.); xiequanliang001@163.com (Q.X.); 2 College of Life Sciences, Ministry of Education Key Laboratory for Ecology of Tropical Islands, Hainan Normal University, Haikou 571158, China; yaoqi7@outlook.com; 3 Institute of Tropical Biosciences and Biotechnology, Chinese Academy of Tropical Agricultural Sciences, Haikou 571101, China

**Keywords:** *Hevea brasiliensis*, calcium-dependent protein kinase, gene family evolution, ethylene signaling, protein–protein interaction network

## Abstract

Natural rubber latex production can be improved by ethylene stimulation in the rubber tree (*Hevea brasiliensis*). However, the expression levels of most functional proteins for natural rubber biosynthesis are not induced after ethylene application, indicating that post-translational modifications, especially protein phosphorylation, may play important roles in ethylene signaling in *Hevea*. Here, we performed a comprehensive investigation on evolution, ethylene-induced expression and protein–protein interaction of calcium-dependent protein kinases (CPKs), an important serine/threonine protein kinase family, in *Hevea*. Nine duplication events were determined in the 30 identified *HbCPK* genes. Expression profiling of *HbCPKs* in three rubber tree cultivars with low, medium and high ethylene sensitivity showed that *HbCPK6*, *17*, *20*, *22*, *24*, *28* and *30* are induced by ethylene in at least one cultivar. Evolution rate analysis suggested accelerated evolution rates in two paralogue pairs, *HbCPK9/18* and *HbCPK19/20.* Analysis of proteomic data for rubber latex after ethylene treatment showed that seven HbCPK proteins could be detected, including six ethylene-induced ones. Protein–protein interaction analysis of the 493 different abundant proteins revealed that protein kinases, especially calcium-dependent protein kinases, possess most key nodes of the interaction network, indicating that protein kinase and protein phosphorylation play important roles in ethylene signaling in latex of *Hevea*. In summary, our data revealed the expression patterns of *HbCPK* family members and functional divergence of two *HbCPK* paralogue pairs, as well as the potential important roles of *HbCPKs* in ethylene-induced rubber production improvement in *Hevea*.

## 1. Introduction

Rubber tree (*Hevea brasiliensis*) is mainly cultivated in the tropical regions, providing the most production of natural rubber, an important industrial material [1]. Ethylene is applied as a routine stimulant to increase the rubber latex yield of rubber tree in agricultural practice for years [2]. However, the molecular mechanism of ethylene signaling of rubber production improvement is not clarified.

Despite the difficulty of molecular genetic research for rubber tree, many works have been performed to investigate important factors in ethylene-induced rubber production improvement (EIRP). Several physiological processes, such as sucrose transport [3], water uptake [4], and synthesis of defense proteins [5] are reported to be activated after ethylene stimulation in *H. brasiliensis* (termed as *Hevea* hereafter). Many functional genes have been identified as to be regulated by ethylene. Sucrose transporter 1A and sucrose transporter 2A can increase sucrose import into laticifers after ethylene stimulation and further increase latex yield [6]. Gene expression analysis showed that ethylene receptor 1, ethylene receptor 2, ethylene insensitive protein 2, and plasma membrane aquaporin were specifically regulated by ethylene treatment [7]. In addition, microRNAs, miR167 (targeting auxin response factor 6) and miR172 (targeting rubber elongation factor 3) were reported to play potential roles in response to ethylene treatment and further improve the rubber latex yield [8]. For the aspect of protein level, various functional proteins involved in natural rubber biosynthesis are not induced upon ethylene treatment, which implies the post-translational modification might be important for EIRP [9]. Our previous work revealed that protein phosphorylation might be important for EIRP [10]. Thus, investigation of protein kinases and the protein phosphorylation catalyzed by kinases in rubber tree will be helpful to better understand the molecular mechanism of EIRP.

Calcium-dependent protein kinase (CPK) belongs to serine/threonine protein kinase family, which contains four domains: N-terminal variable domain, serine/threonine kinase domain, auto-inhibitory junction domain and EF-hand calcium binding domain [11,12,13]. N-terminal variable domain is highly variable in both length and sequences, and contains myristoylation and palmitoylation sites, which contribute to membrane localization [12]. Auto-inhibitory domain serves as a pseudo-substrate and blocks the activity of the enzyme in the absence of Ca^2+^ stimulation with CaM-LD binding site [14]. The EF-hand calcium binding domains hold up four elongation factor hands and CPKs in monocot and dicot plants possess at least four D-x-D and two D/E-E-L motifs in their EF-hands, that bind the calcium icons and play a significant role in gene regulation [12,14]. Depending on the unique structure, a kinase domain with an EF-hand containing regulatory domain, CPKs can respond diverse endogenous and environmental cues and translate Ca^2+^ signals into downstream protein phosphorylation [15]. CPKs play important roles in many aspects of plant growth and development and in responding to various stimulate including biotic and abiotic stresses and hormones [16].

Genome-wide investigation of *CPK* gene family have been reported in *Arabidopsis* (*Arabidopsis thaliana*), rice (*Oryza sativa*), soybean, maize and grape, as well as cassava (*Manihot esculenta*), which is closely related to *Hevea* [12,16,17,18,19,20]. The *HbCPK* gene family has been reported recently, identifying 30 *HbCPKs* and profiling their expression patterns in six plant species using next-generation sequencing data [21]. However, the evolutionary and syntenic relationships of *HbCPK* members, as well as the protein expression levels of CPK family members in response to ethylene treatment were not thoroughly analyzed. Here, we performed a comprehensive evolutionary and syntenic analysis of *HbCPK* genes. The expression profiles of *HbCPKs* in different tissues and after ethylene treatment in three varieties were analyzed by quantitative real-time PCR (qRT-PCR) and semi-quantitative PCR. The protein–protein interaction (PPI) network of ethylene-response proteins were further analyzed, suggesting that the kinase protein members and phosphorylated proteins were involved in the key nodes of the ethylene-induced protein interaction network clusters. Our work provides a more comprehensive investigation of the evolutionary relationships, expression patterns and PPI relationships of *CPK* members in ethylene signaling in *Hevea*, as well as potential transgenic targets of molecular breeding of rubber tree for improving natural rubber production in the future.

## 2. Results

### 2.1. Identification and Characteristics of CPK Members in H. brasiliensis

A BLASTP search for *Hevea* genome database was performed using *Arabidopsis* and rice CPK protein sequences, a total of 41 genome-deduced proteins were identified as HbCPK candidates. Only sequences containing both protein kinase domain (PF00069) and EF-hand_7 (PF13499) domain were confirmed as CPKs, resulting in 30 HbCPK family members (Table S1). The open reading frame lengths of the 30 identified *HbCPKs* range from 1308 bp to 1896 bp, and the calculated protein molecular weights range from 49.19 to 70.79 kDa and isoelectric points range from 5.14 to 9.25.

The 30 *HbCPK* members were distributed onto 30 different scaffolds (Figure S1). Nine duplicated events were identified (*HbCPK1*/*HbCPK26*, *HbCPK2*/*HbCPK7*, *HbCPK3*/*HbCPK8*, *HbCPK4*/*HbCPK11*, *HbCPK13*/*HbCPK30*, *HbCPK16*/*HbCPK28*, *HbCPK18*/*HbCPK9*, *HbCPK19*/*HbCPK20* and *HbCPK21*/*HbCPK22*). The conserved domains were shown in multiple sequence alignment of HbCPKs (Figure S2). In addition, 12 conserved motifs were predicted using Multiple Em for Motif Elicitation (MEME, version 4.12.0) (http://meme-suite.org/doc/authors.html?man_type=web) program (Figure S3). Motif 3, 7, 5, 8 and 9 represent protein kinase domain, auto-inhibitory domain and four EF-hand domains, respectively. A phylogenetic tree was constructed using the CPK protein sequences of *Arabidopsis*, *O. sativa*, *M. esculenta* and *Hevea*, dividing the CPKs into four groups (Group I to IV), containing eleven, eight, eight and two members, respectively (Figure S4).

### 2.2. Gene Structure, Duplication Events and Syntenic Analysis

**Figure 1:**
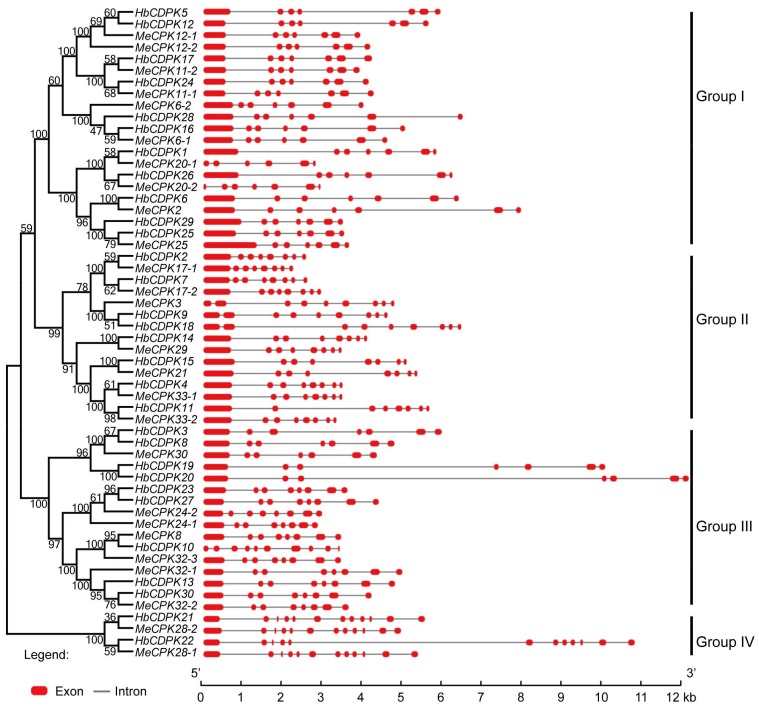
Phylogenetic and gene structure analysis of *CPKs*. Exon-intron distribution was analyzed using GSDS software 2.0 (http://gsds.cbi.pku.edu.cn/). Tree-view is on the left, exon-intron distribution is on the right side, introns and exons are represented by black lines and red boxes, respectively, and the genomic length is indicated at the bottom.

The exon-intron distribution reveals the evolutionary relationship of gene families [22]. Gene structure analysis of *HbCPKs* showed that the members in same subfamily shared similar distribution pattern in both numbers and length of exons and introns. As shown in Figure 1, group I contains 6–7 exons, group II contains 7–12 exons, group III contains 7–8 exons and group IV contains 12 exons. Moreover, the paralogue pairs of *HbCPK3*/*HbCPK8, HbCPK13*/*HbCPK30*, *HbCPK18*/*HbCPK9* and *HbCPK19*/*HbCPK20* were determined as rubber tree specific duplicated events by comparing the phylogenetic relationship and exon-intron distribution patterns of *HbCPKs* and that of *MeCPKs*.

**Figure 2:**
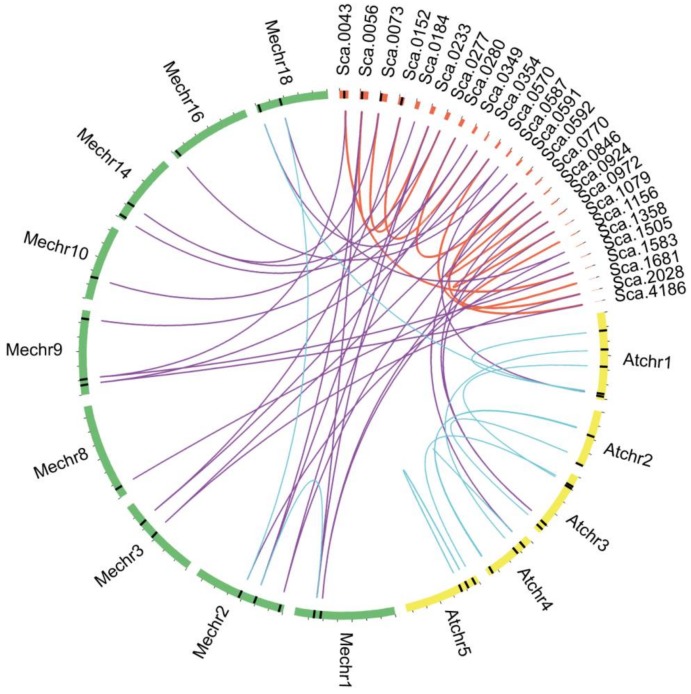
Segmental duplication of *HbCPK* genes and syntenic analysis of *H. brasiliensis*, *M. esculenta* and *A. thaliana CPKs*. Chromosomes and scaffolds are shown in different colors in circular form. The positions of the *CPK* genes are marked with black lines on the circle. The duplicated *CPK* pairs in *H. brasiliensis* are linked by red lines and syntenic relationships between *H. brasiliensis* and the other two species are linked by purple lines, while blue lines indicate the *CPK* pairs between or inside *M. esculenta* and *A. thaliana*.

The syntenic analysis of *Hevea* was performed using gene location information of *CPKs* in *Arabidopsis* and a closely related spurge plant cassava to investigate the expansion of *HbCPK* family. No tandem duplication events were discovered because of the limitation of chromosomal information of the current version of rubber tree genome assembly. Nine segmental duplication events were identified and the paralogue gene pairs were then defined as syntenic genes (Figure 2).

The *Ka*/*Ks* ratio was calculated to assess the selection pressure for each duplicated *HbCPK* paralogue pairs. The results showed that all paralogues were under purifying selection pressure (Table 1). Meanwhile, Tajima relative rate was used to estimate the evolutionary rate of these duplication pairs. The *HbCPK9*/*HbCPK18* and *HbCPK19*/*HbCPK20* duplication pairs have a prominently accelerate evolutionary rates (Table 2).

### 2.3. Tissue-Specific and Ethylene-Induced Expression Analysis of HbCPKs in Three Rubber Tree Varieties

**Figure 3:**
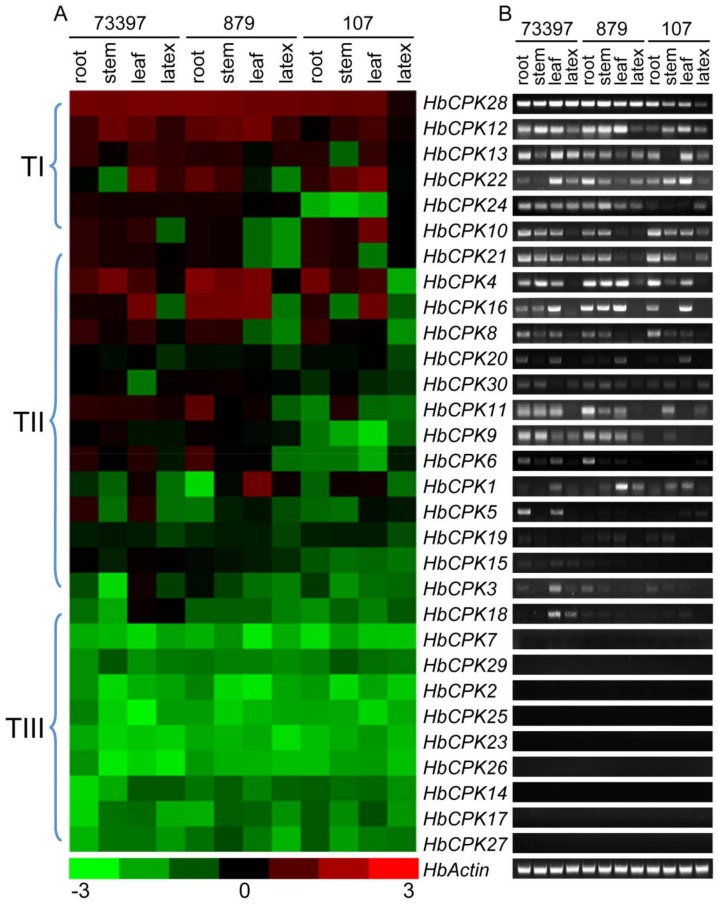
Expression profiles of *HbCPKs* in different tissues obtained from three cultivars. Expression patterns of *HbCPKs* were determined by qRT-PCR (**A**) and semi-quantitative PCR (**B**). The *HbCPK* genes were clustered by their expression patterns (left). *HbActin* was used as reference gene. The semi-quantitative PCR results were shown in electrophoretogram (**B**).

Root, stem, leaf and latex of three cultivated rubber tree varieties (Reyan8-79 with high yielding, low ethylene sensitivity; Reyan7-33-97 with medium yielding and medium ethylene sensitivity; PR107 with low yielding and high ethylene sensitivity) were used in this study to provide better understanding of the roles of *HbCPKs*. The expression pattern of *HbCPKs* was visualized using qRT-PCR (Figure 3A) and semi-quantitative PCR assays (Figure 3B) to analyze the tissue specific expressions. Gene-specific primers used in PCR assays are listed in Table S2. The expression pattern can be divided into three clusters: *HbCPK12*, *13*, *21*, *22*, *24* and *28* of cluster TI mainly expressed in all four tissues; *HbCPK1*, *3*, *4*, *5*, *6*, *8*, *9*, *11*, *15*, *16*, *18*, *19*, *20*, and *30* of cluster TII mainly expressed in roots, stems and leaves of Reyan7-33-97 and Reyan8-79; *HbCPK2*, *7*, *14*, *17*, *23*, *25*, *26*, *27* and *29* of cluster TIII could not be detected. The members of *HbCPK12*, *HbCPK13*, *HbCPK21*, *HbCPK22*, *HbCPK24* and *HbCPK28* showed high expression levels in latex, indicating their potential function in rubber latex production. Notably, paralogue pairs with accelerated evolutionary rates showed divergent expression patterns in different tissues (*HbCPK9*/*HbCPK18* and *HbCPK19*/*HbCPK20*), suggesting their potential functional divergence after duplication events (Figure 3).

**Figure 4:**
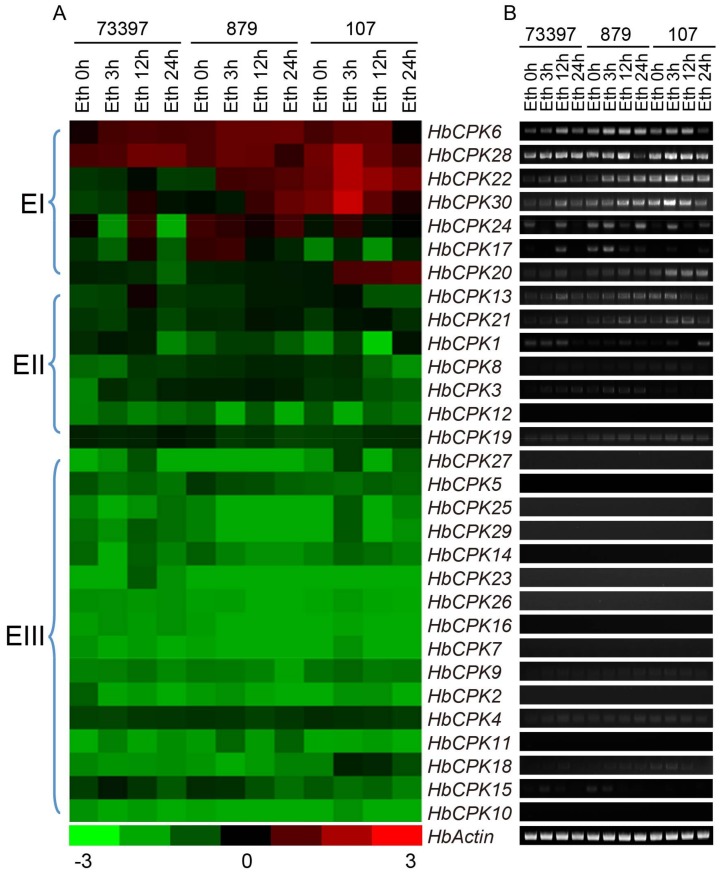
Expression profiles of the 30 *CPK* genes in latex from three rubber tree varieties upon ethylene stimulation. (**A**) The heat map for 30 *HbCPKs* at 0, 3, 12 and 24 h under ethylene stimulate. The color bar shows at the bottom. The genes were ordered by the cluster of their expression patterns (left); (**B**) electrophoretogram of semi-quantitative PCR under different abiotic stresses. *HbActin* serves as the reference gene.

The expression levels of *HbCPKs* were further examined in the latex of three cultivars after ethylene treatment for 0, 3, 12 and 24 h. The results showed that *HbCPK13*, *HbCPK21* and *HbCPK28* were significantly induced after ethylene stimulation in all three cultivars. Meanwhile, the expression levels of *HbCPK20*, *HbCPK22* and *HbCPK30* were specifically promoted by ethylene stimulation in the ethylene-sensitive cultivar PR107 (Figure 4). Moreover, *HbCPK6* and *HbCPK30* which have very low expression levels in latex before ethylene treatment showed significantly up-regulated after ethylene stimulation.

### 2.4. Cis-Element Distribution Analysis of Promoter Regions in HbCPKs

**Figure 5:**
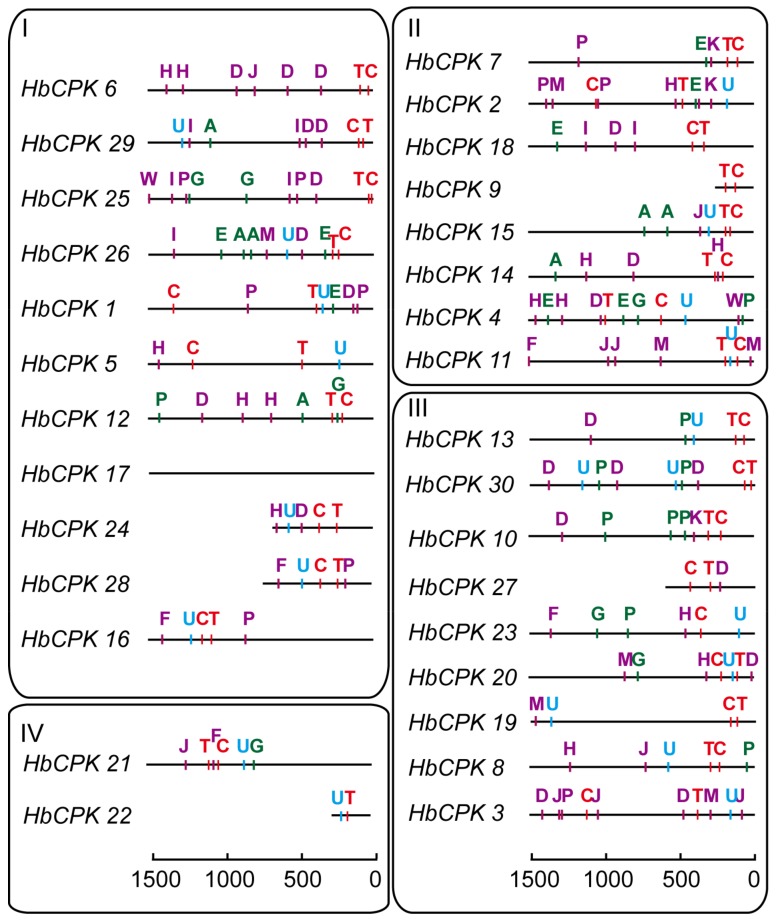
Distribution of *cis*-elements in *HbCPK* gene promoter regions. The *cis*-elements of the 1.5 kb up-stream promoter regions of *HbCPKs* were predicted using PlantCARE software. The *HbCPKs* were divided into four groups according to the phylogenetic sub-families (Group I–IV). Colored capital letters represent different *cis*-elements at corresponding positions. Red, green, purple and blue letters represent transcription initiation, phytohormone response, stress response and high transcription classification of *cis*-elements, respectively. The detailed information for each *cis*-element is provided (Table S3).

The distribution of *cis*-elements in the promoter regions of *HbCPKs* was analyzed to investigate the potential regulation factors of *HbCPK* family members. The *cis*-elements were symbolized by capital letters with different colors and detailed information was also supplied (Figure 5, Table S3). The results showed that the promoter regions in *HbCPK1*, *HbCPK2*, *HbCPK4*, *HbCPK7*, *HbCPK18* and *HbCPK26* have one or two ethylene response *cis*-elements, suggesting the potential regulation of these genes under ethylene. Notably, the *cis*-element distribution in promoters of paralogues *HbCPK9*/*18* and *HbCPK19*/*20* with accelerated evolutionary rates are various, providing further evidence that these two paralogue pairs have different regulation patterns and are ongoing functional divergence.

### 2.5. Protein Expression and PPI Analyses of Ethylene-Induced Proteins in Latex

**Figure 6:**
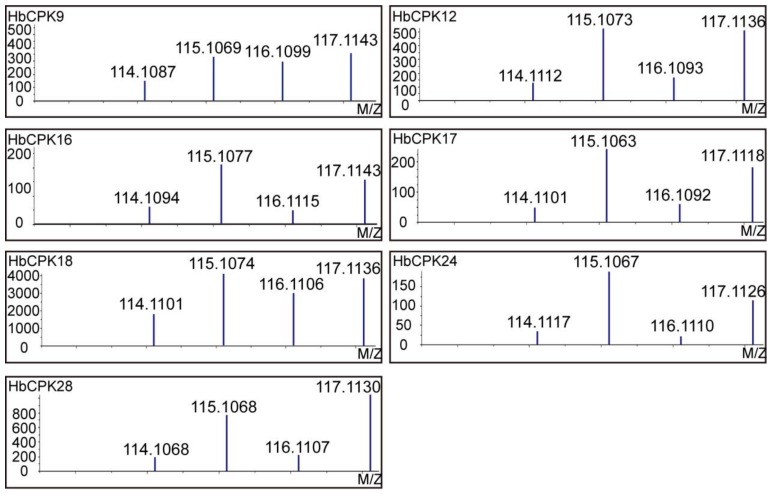
Proteomic validation of *HbCPK* expression patterns in rubber tree latex before and after ethephon treatment. Representative mass spectra indicating the signal intensities of isobaric tags for the validated CPKs. The spectra correspond to the peptides of “SGYITMEELEHALK”, “LSRLK”, “LYQGYTQPEEQSTASHTKR”, “IGQGQFGTTYLCTNK”, “GQFGVTYLVTHK”, “QATSSSSSTTKPANTVLPYQTSR” and “IIVGVVEACHSLGVMHR” that mapped to HbCPK9, HbCPK12, HbCPK16, HbCPK17, HbCPK18, HbCPK24 and HbCPK28, respectively.

To investigate the protein expression pattern of HbCPKs after ethylene stimulation, our previous isobaric tags for relative and absolute quantification (iTRAQ) proteomic data [10] were re-analyzed using *Hevea* genome-derived protein database, identifying 3902 high-quality proteins (95% confidential peptides ≥2; Table S4). Seven HbCPK members were detected, of which six (HbCPK12, HbCPK16, HbCPK17, HbCPK18, HbCPK24 and HbCPK28) were significantly induced by ethylene (fold change ≥2; Table 3). The raw tandem mass spectrometry (MS/MS) spectra mapped to HbCPK proteins were provided (Figure S5). The representative signal intensities of iTRAQ report groups of seven identified HbCPK members were shown (Figure 6). These data further demonstrated that HbCPK members could be significantly induced upon ethylene treatment not only in transcript level but also in protein level.

**Figure 7:**
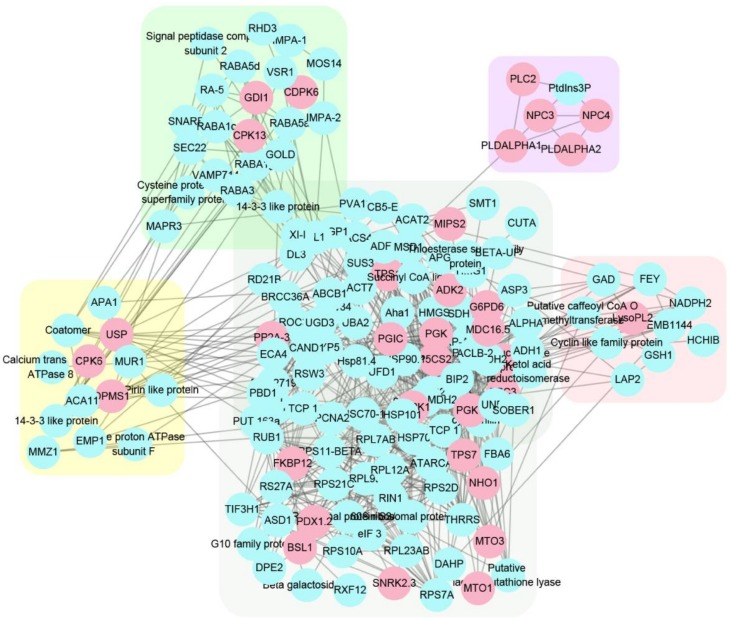
PPI networks of ethylene-regulated latex proteins and ethylene-responsible phosphorylated proteins. The 492 ethylene-regulated and 59 phosphorylated proteins identified were analyzed using the STRING database 10.5 with a medium confidence (0.400). Blue circles represent individual PPI network nodes with protein names. Pink circles indicate protein kinases. Five network clusters were shadowed by different colors.

Further comparative proteomics analysis of iTRAQ data resulted in 492 significantly different abundant proteins (DAPs). Protein–protein interaction analysis of these DAPs and 59 previously reported phosphorylated proteins [10] was performed using STRING 10.5. The result exhibited five PPI network clusters which were shadowed with different colors in Figure 7. Notably, protein kinases, especially calcium-dependent protein kinases, possess most key nodes of the interaction network (pink circles in Figure 7), implying the crucial roles of kinase proteins and protein phosphorylation in EIRP. These data for the first time validated the importance of HbCPK members in EIRP at protein level.

## 3. Discussion

Ethylene stimulation is a widely used technological that can improve nature latex production [23]. Paradoxically, the expression levels of genes that known to involve in rubber biosynthesis are not upregulated upon ethylene stimulation. Most rubber latex biosynthesis related proteins, such as farnesyl diphosphate synthase [24], rubber elongation factor and small rubber particle protein [25], are not induced by ethylene stimulation, indicating that post-translational modification might be important for ethylene treatment response in rubber tree. Our previous works discovered that phosphorylation level of the latex proteins, and the expression level of calcium-dependent protein kinase was significantly increased after ethylene stimulation [10]. Xiao et al. identified 30 *HbCPKs* and analyzed their expression patterns in *Hevea* and five other plant species using Solexa sequencing data, however, the transcript and protein expression levels of CPK family members in response to ethylene treatment were not thoroughly analyzed [21]. Hence, we performed a thoroughly investigation on the gene expression pattern and PPI network of *CPK* members after ethylene treatment. Our study provided new evidence to support the hypothesis that *HbCPK* is involved in the ethylene induced rubber latex production.

Gene duplication may cause functional diversity and further altered expression profiles and protein properties, and is a major evolutionary driver for helping plant species to meet a broader array of lineage-specific requirements [26]. The numbers of 3, 9, 2, 10 duplication events were found in soybean, rice, grape, and maize genome [17,18,19,20]. Analysis of *Ka*/*Ks* selection pressure showed that all *HbCPK* paralogues were under purifying selection (Table 1), suggesting that the conservation of *HbCPK* proteins might be crucial for their functions plant development. The expansion of a gene family within a genome could occur through gene functional divergence after duplication events. The *HbCPK9*/*HbCPK18* and *HbCPK19*/*HbCPK20* duplication pairs had various expression patterns in different tissues and after ethylene stimulation, as well as different *cis*-elements distribution patterns (Figure 3, Figure 4 and Figure 5), providing further evidence of the accelerated evolutionary rate of these two paralogues in rubber tree. As shown, *HbCPK20* was mainly up-regulated during ethylene stimulate in the latex of PR107 (high ethylene sensitivity cultivar) while *HbCPK19* was not (Figure 4), suggesting that *HbCPK19* might play the same role as its *Arabidopsis* orthologue *AtCPK13* (regulating the stomatal aperture) [27] and *HbCPK20* might gain new functions in EIRP of rubber tree.

The expression levels of some genes were found to be inconsistent in latex and 0 h ethylene treated latex, which may be caused by different experimental materials used in Figure 3 and Figure 4. Similar results were also reported by Xiao et al. [21]. However, this difference in expression levels does not affect the analysis on *HbCPKs* response to ethylene stimulation. Besides, *HbCPK1*, *2*, *4*, *7*, *18* and *26* had one or two ethylene responsive *cis*-element (Figure 5), but the expression levels of these genes were not induced by ethylene stimulation (Figure 4). The inconsistency between *cis*-element predicted by software and the regulation of the gene expression was reported by many researches [28,29]. It suggested that our understanding of the regulation mechanism of gene expression is still poor and the *cis*-elements predicted by the sequence information may have no function in many cases.

Introduction of genome predicated protein database to analyze our previous proteomic data greatly improved the number of identified proteins from 1596 to 3902 (Table S4). For the first time, seven HbCPK members were identified at protein level with raw spectra provided, in which HbCPK12, 16, 17, 18, 24 and 28 were significantly induced by ethylene (Table 3, Figure 6 and Figure S5), but the expression of *HbCPK12*, *16* and *18* were not induced in mRNA levels after ethylene stimulation (Figure 4). Generally, frequent transcriptome and high mRNA stability can improve protein accumulation. However, many factors participate in the processes from transcription to translation and affect the translation efficiency of a given mRNA. The poly (A) tail length of the mRNA affects transcript stability and is closely correlated with translational efficiency [30,31]. This phenomenon could partly explain the different expression changes in response to ethylene treatment at cDNA and protein level we observed in Figure 4 and Figure 6. The fact that protein kinases possess the most key nodes in the PPI network suggested that ethylene signaling promotes the natural rubber production mainly through phosphorylation of functional proteins, but not through increasing the accumulation of functional enzymes (Figure 7). These proteomic analyses provide an explanation of the phenomenon that natural rubber biosynthesis related proteins are not induced by ethylene signal.

In summary, our research provides a comprehensive evolutionary and syntenic analysis of *HbCPK* members, suggesting that all *HbCPKs* were under purifying selection pressure and two duplication paralogues had an accelerated evolutionary rate. The expression profiling of *HbCPK* family demonstrated that the ethylene-induced *HbCPK* members have potential diverse functions in EIRP in three different ethylene-sensitivity rubber tree cultivars. The PPI network of DAPs revealed that protein kinases and phosphorylated proteins were involved in the key nodes of the ethylene-induced protein interaction network clusters, indicating the potential interaction among protein kinases and protein phosphorylation regulated upon ethylene stimulation in latex. This work provides a more comprehensive understanding of the *HbCPKs* in ethylene induced rubber production improvement and potential transgenic targets for future molecular breeding of rubber tree for improving natural rubber production.

## 4. Materials and Methods

### 4.1. Identification and Sequence Analyses of CPK Gene Family in Rubber

Protein sequences of CPK family members in *Arabidopsis* (*Arabidopsis thaliana*) and rice (*Oryza sativa*) were used for query [12,17]. Local BLASTP search was performed to identify CPK members in rubber tree genome [32]. All the remaining CPK candidates were further verified by InterProScan server (http://www.ebi.ac.uk/interpro/) to assess the existence of protein kinase domain (PF00069) and EF-hand_7 domain (PF13499). A carefully manual review of these sequences was carried out to amend the underlying mistake of rubber tree genome database.

The genomic location of *HbCPKs* was performed using local BLASTN against rubber tree genome database. Mapinspect was used to draw the location of *CPKs* in different scaffolds. The amino acid sequences of CPKs in rubber tree, *Arabidopsis*, rice, and a related-species cassava were used to construct phylogenetic tree by MEGA 5.0 (Hachioji, Tokyo, Japan) using neighbor-joining method with 1000 bootstrap test. Multiple sequence alignment was conducted using ClustalX 2.0 (Belfield, Dublin, Ireland) with default parameters to determine the conserved domain of CPKs. Multiple Em for Motif Elicitation (MEME) online software (http://meme-suite.org/tools/meme) was used to identify the conserved motifs of HbCPK members. The molecular weight and isoeletric points of HbCPKs were predicted by ExPASy server (http://expasy.org/). Softberry (http://linux1.softberry.com/berry.phtml) was used to predicted subcellular location of HbCPK members.

### 4.2. Gene Structure, Duplication Event and Syntenic Analysis

Gene structure was analyzed using GSDS server (http://gsds.cbi.pku.edu.cn/) based on the open reading farm (ORF) against *HbCPKs* and *MeCPKs* genomic sequences. Paralogues was determined by multiple sequence alignment with the identification > 90% [33]. DnaSP 5.0 software (Barcelona, Catalonia, Spain) was used to calculate *Ka* (nonsynonymous substitution rate) and *Ks* (synonymous substitution rate). The *Ka*/*Ks* ratios for the *HbCPK* paralogues were calculated to assess the selection pressure; the ratio >1, <1, or =1 indicates positive, negative or neutral evolution, respectively [29]. Tajima relative rate tests [34] were analyzed by MEGA 5.0 using the amino acid sequences of the duplicated *HbCPK* pairs. The syntenic relationships of paralogues and/or orthologues among *Arabidopsis*, cassava and rubber tree were analyzed using the Circos program [35].

### 4.3. Plant Material and Ethylene Treatment

Rubber trees of the three cultivars, named Reyan8-79 (high yielding, low ethylene sensitivity), Reyan7-33-97 (medium yielding, medium ethylene sensitivity) and PR107 (low yielding, high ethylene sensitivity) were planted in the experimental farm of Chinese Academy of Tropical Agricultural Sciences in Hainan Province in China. Different tissues, including roots, stems, leaves and untreated latex were collected from 10-year-old mature trees. Ethylene treated latex were collected from these three cultivars 0, 3, 12 and 24 h after application of 1.5% ethephon. All materials were frozen in liquid nitrogen immediately after collected and stored at −80 °C.

### 4.4. Gene Expression Profiling of HbCPKs

Total RNAs were extracted using RNAprep pure plant kit (TIANGEN, Beijing, China) from different rubber tree materials, and 1 μg of total RNA was used for first-strand cDNA synthesis. The gene-specific primers for qRT-PCR were designed using the software Primer Primer 5.0 (Davis, CA, USA) according to *HbCPK* gene sequences listed in Supplementary Table S1. Rubber tree *actin* gene (GenBank Acc. HQ260674.1) was used as an internal control. Quantitative real-time PCR (qRT-PCR) was conducted using Mx3005P real-time PCR system with SYBR green master mix (2X) (Thermo Fisher Scientific, Foster, CA, USA) in accordance with previously described PCR conditions [36]. Data were processed using 2^−ΔΔ*C*t^ methods, and the relative expression level of each *CPK* gene were used to generate a heat map using MultiExperiment viewer software (MeV, version 4.9, Boston, MA, USA). Semi-quantitative PCR assays were performed using the same cDNA templates and primers used for qRT-PCR. The expression levels of each *HbCPK* gene were visualized using agarose gel electrophoresis of the corresponding PCR products.

### 4.5. Cis-Element Distribution Analysis in HbCPK Promoter Regions

The 1500 bp sequence of the genomic sequence upstream to the start codon of each gene was obtained from genome database to investigate the *cis*-element distribution in *HbCPK* promoter regions. These sequences were then submitted to PlantCARE server [37] to predict putative *cis*-element.

### 4.6. Interaction Network Analysis of Ethylene-Induced Proteins in Latex

The ethylene-induced and phosphorylated latex proteins were obtained by re-analysis of comparative proteomics data of rubber latex after ethylene treatment, using Proteinpilot 5.0 (Redwood City, CA, USA) and *Hevea* genome-derived protein database [10,30]. Only proteins with 95% confidential peptides ≥2 were considered as identified (Proteinpilot score ≥1.3). The PPI network was constructed by Search Tool for the Retrieval of Interacting Genes (STRING10.5) [38] with the confidence score ≥0.400, and further visualized using Cytoscape 3.6.0 [39].

## Figures and Tables

**Table 1:** The *Ka*/*Ks* ratios for duplicate CPK genes in *H. brasiliensis*.

Paralogous Genes	*Ka*	*Ks*	*Ka*/*Ks*	Selective Pressure
*HbCPK16–HbCPK28*	0.0129	0.1660	0.0777	Purifying selection
*HbCPK1–HbCPK26*	0.0276	0.2004	0.1377	Purifying selection
*HbCPK3–HbCPK8*	0.0193	0.2290	0.0843	Purifying selection
*HbCPK13–HbCPK30*	0.0274	0.1840	0.1489	Purifying selection
*HbCPK4–HbCPK11*	0.0308	0.2527	0.1219	Purifying selection
*HbCPK9–HbCPK18*	0.0434	0.1876	0.2313	Purifying selection
*HbCPK2–HbCPK7*	0.0227	0.1941	0.1170	Purifying selection
*HbCPK21–HbCPK22*	0.0169	0.2004	0.0843	Purifying selection
*HbCPK19–HbCPK20*	0.0214	0.1767	0.1211	Purifying selection

*Ka*: non-synonymous substitution rate; *Ks*: synonymous substitution rate.

**Table 2:** Tajima relative rate tests of *CPK* gene pairs in rubber tree ^a^.

Testing Group	Mt ^b^	M1 ^c^	M2 ^d^	Χ^2^	*P* ^e^
*HbCPK4/HbCPK11* with *MeCPK33-2*	489	20	13	1.48	0.22302
*HbCPK9/HbCPK18* with *MeCPK3*	457	7	47	29.63	0.00001
*HbCPK16/HbCPK28* with *MeCPK6-1*	529	8	7	0.07	0.79625
*HbCPK26/HbCPK1* with *MeCPK20-1*	250	5	11	2.25	0.13361
*HbCPK3/HbCPK8* with *MeCPK30*	514	7	16	3.52	0.06057
*HbCPK30/HbCPK13* with *MeCPK32-2*	485	10	17	1.81	0.17793
*HbCPK19/HbCPK20* with *MeCPK30*	370	6	0	6.00	0.01431
*HbCPK2/HbCPK7* with *MeCPK17-2*	465	16	9	1.96	0.16151
*HbCPK22/HbCPK21* with *MeCPK28-1*	507	17	10	1.81	0.17793

^a^, the Tajima relative rate test was used to examine the equality of evolutionary rate between rubber tree paralogues; ^b^, Mt is the sum of the identical sites in all three sequences tested; ^c^, M1 is the number of unique differences in the first paralog; ^d^, M2 is the number of unique differences in the second paralog; ^e^, if *p* < 0.05, the test rejects the equal substitution rates between the two duplicates and infers that one of the two duplicates has an accelerated evolutionary rate.

**Table 3:** Identification and quantitative information of seven detected HbCPKs.

HbCPK	Peptide	H_2_O-48 h	Eth-48 h	H_2_O-96 h	Eth-96 h
	Numbers	(114)	(115)	(116)	(117)
HbCPK9	14	1.0000 *	1.0471	1.0186	1.0965
HbCPK12	2	1.0000	3.3712	1.1478	3.1326
HbCPK16	6	1.0000	4.0926	2.1478	4.4463
HbCPK17	9	1.0000	9.7351	1.2985	9.1039
HbCPK18	7	1.0000	2.3335	0.8017	2.1677
HbCPK24	8	1.0000	13.9211	1.6346	12.5966
HbCPK28	6	1.0000	4.1687	1.7061	5.2481

* Relative protein expression levels are defined as the ratio of 114/114, 115/114, 116/114 and 117/114. Eth, ethylene.

## Supplementary Materials

Supplementary materials can be found at http://www.mdpi.com/1422-0067/19/4/947/s1.
