# Supplementary material for: Calcium-Dependent Protein Kinase Family Genes Involved in Ethylene-Induced Natural Rubber Production in Different Hevea brasiliensis Cultivars
Source: Int J Mol Sci. 2018 Mar 22;19(4):947. doi: 10.3390/ijms19040947 (PMC5979512; doi:10.3390/ijms19040947)
Supplement: Supplementary file 1 [file ijms-19-00947-s001.zip › Supplementary files/Supp_Figures.pdf]

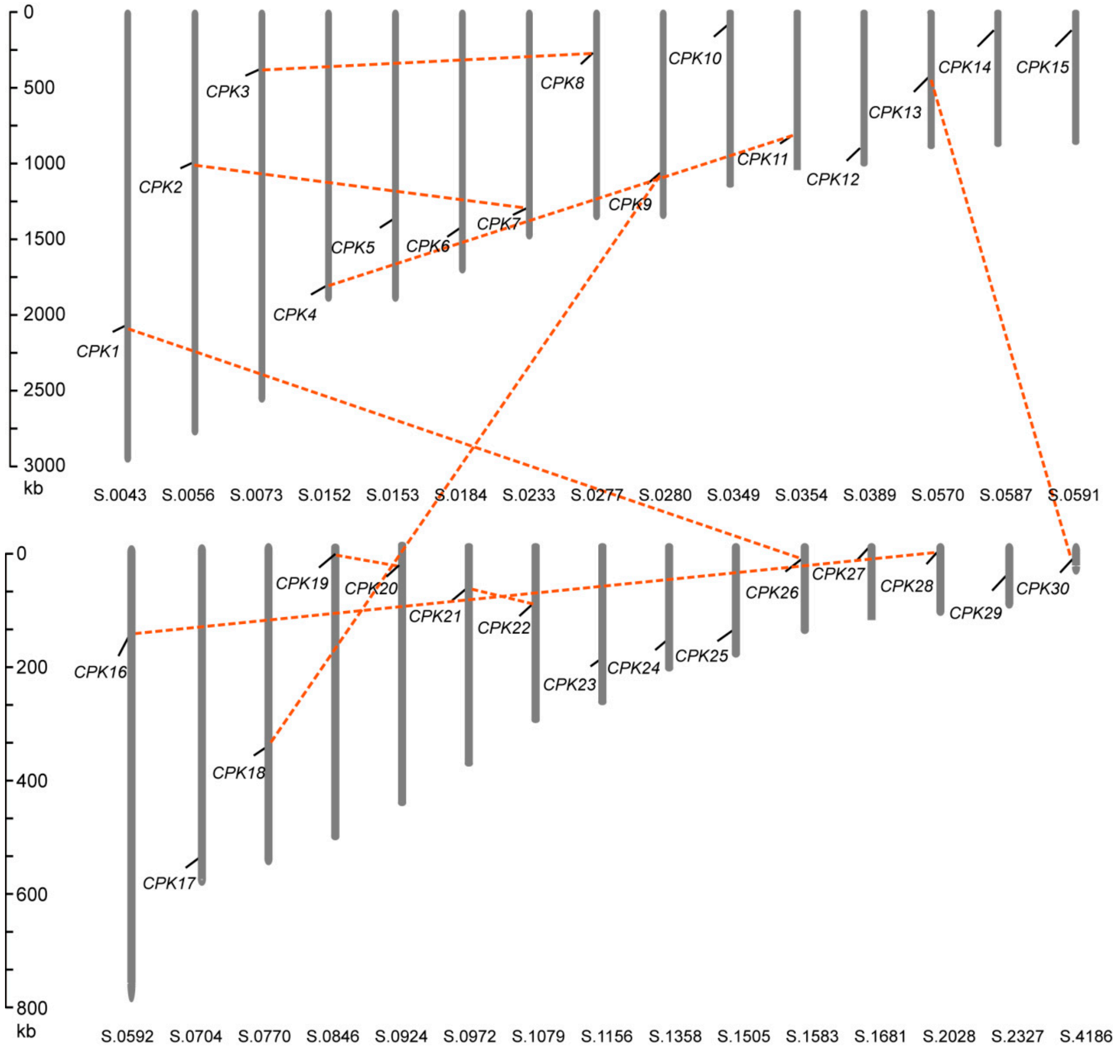

Figure S1. Scaffold distribution of *CPK* genes in *H. brasiliensis*. The *HbCPK* genes were located to 30 individual scaffolds. Vertical columns represent the scaffolds with scaffold numbers at the bottom and scale bars on the left. Paralogs were connected with red spotty lines.

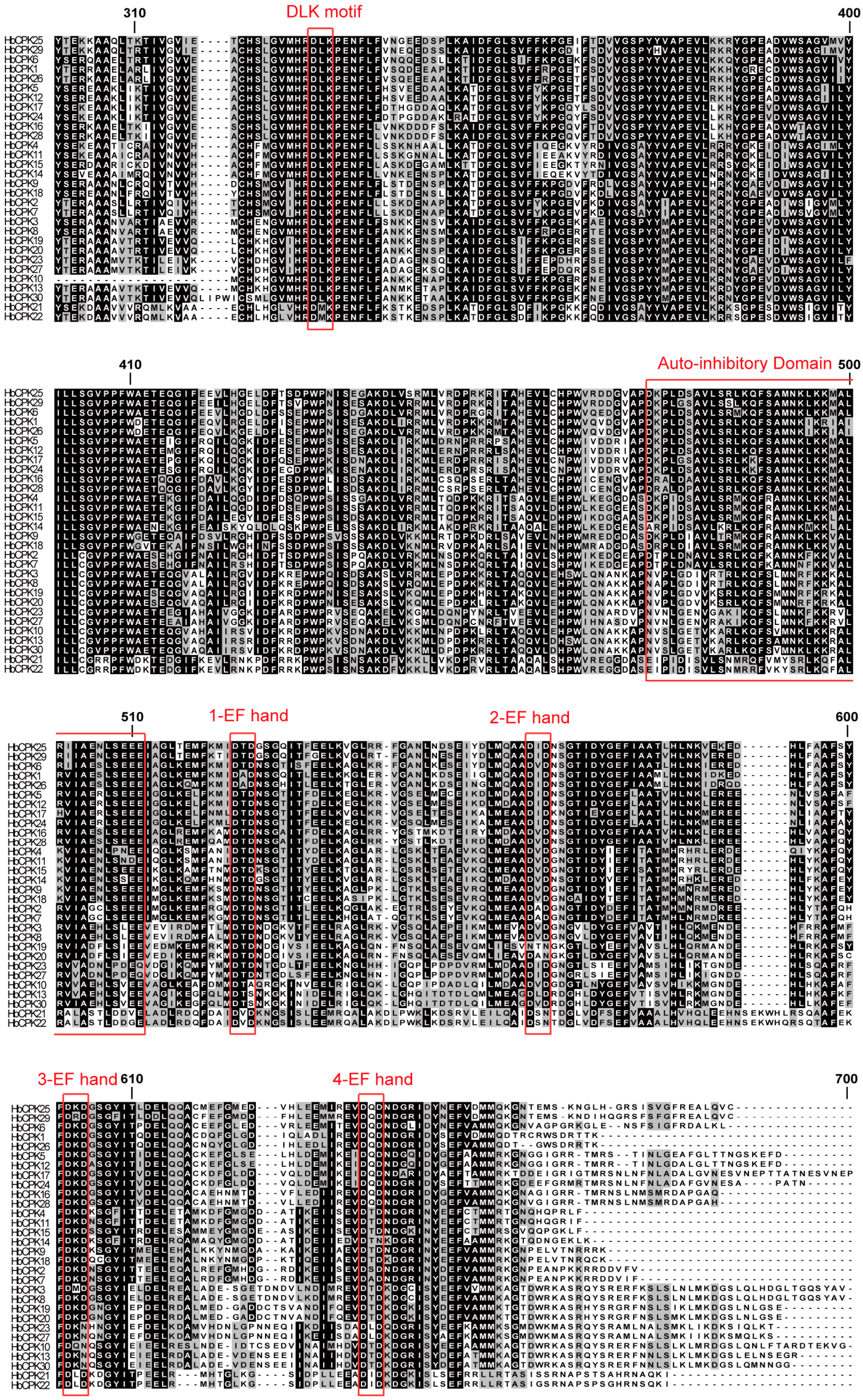

Figure S2. Protein sequence alignments of the CPKs in *H. brasiliensis*. Sequence alignment was performed using ClustalW program. The conserved region is covered by shadow. The alignment region ranged from 300 to 700 amino acids, each line has 100 amino acids. The conserved domains are marked in red boxes.

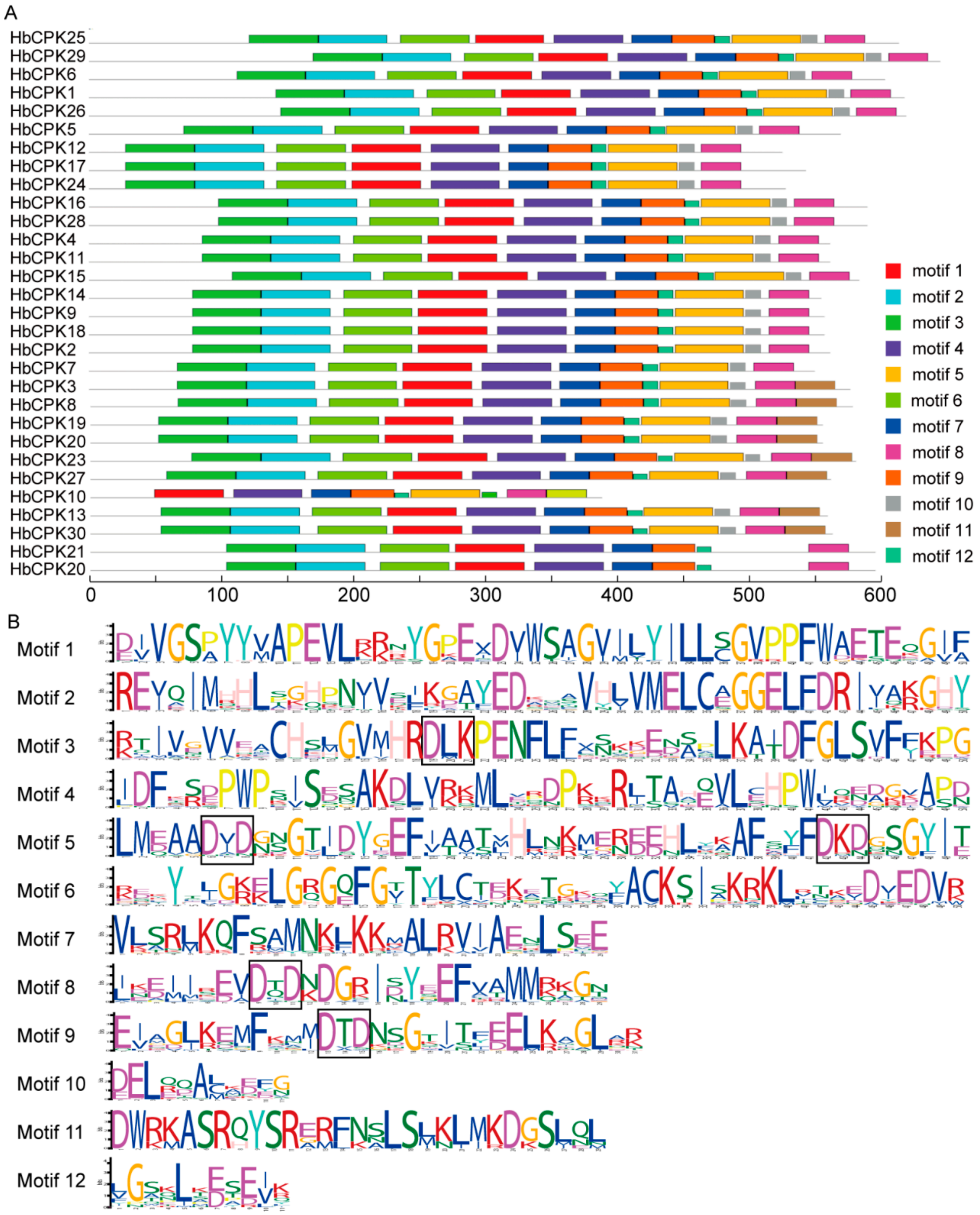

Figure S3. Motif analysis of CPK gene family in *H. brasiliensis*. The HbCPK protein sequences were used to identify conserved motif in rubber trees by MEME program. A. Twelve motifs distribution of HbCPKs. The colored rectangles represent 12 conserved motifs, respectively. B. Motif logo. shows the amino acid sequence of these 12 motifs, the height of each letters shows the frequency of amino acids at that site, the black frames are key sites of the five conserved motifs.

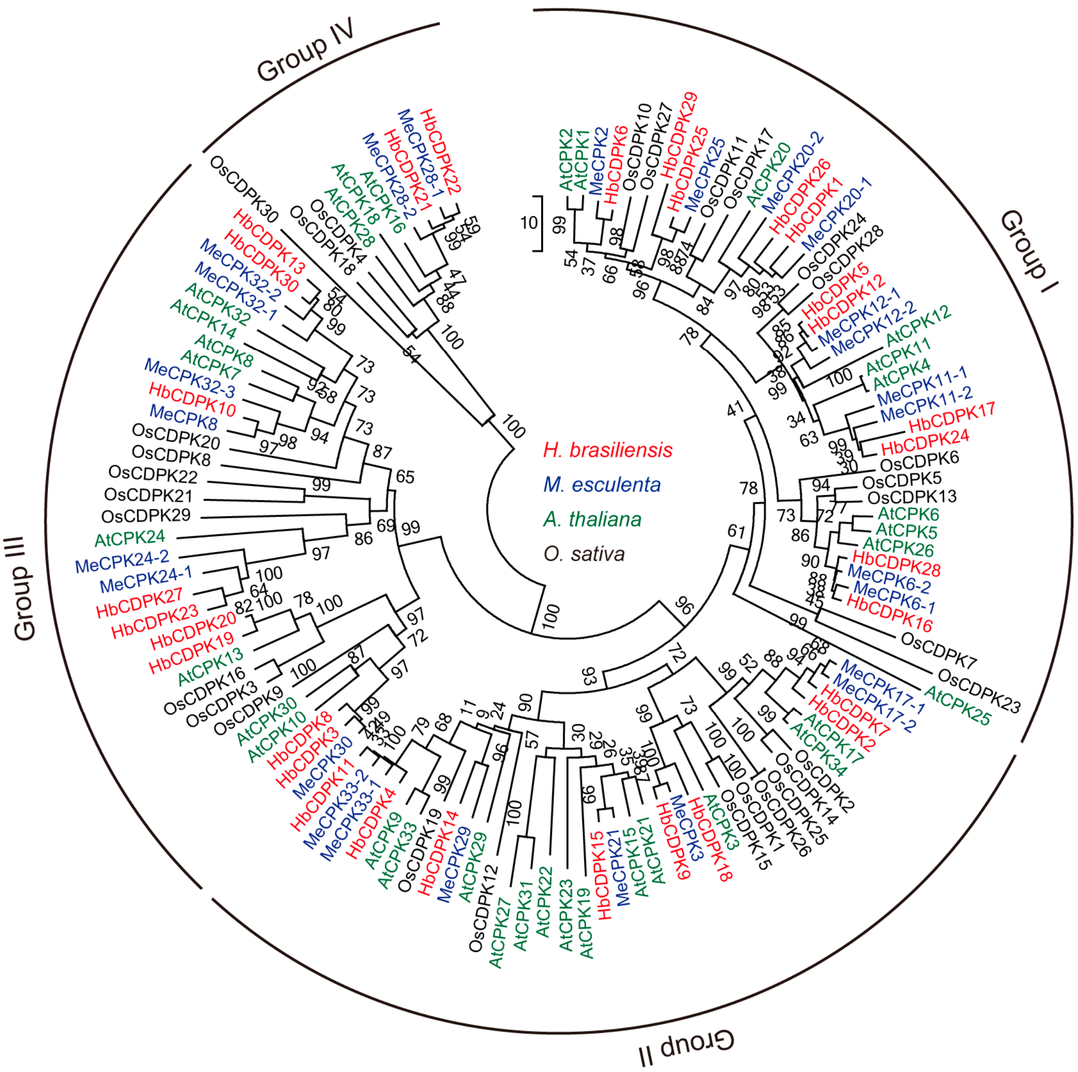

Figure S4. Phylogenetic tree of the CPK gene family in four species. Phylogenetic tree was constructed by ClustalX 2.0 and MEGA5.0 software with neighbor joining (NJ) method and 1000 bootstrap replicates. CPKs from four species are shown in different colors and four colored sectors indicated four distinct clades (Group I–IV).

A

MS/MS spectrum mapped to HbCPK9

MS/MS for HbCPK9 peptied: AGLPK

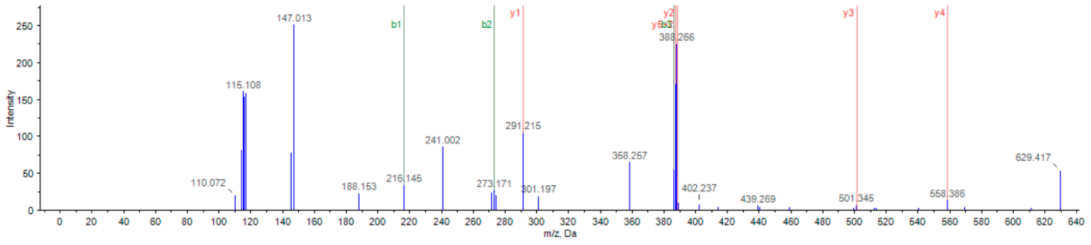

MS/MS for HbCPK9 peptied: DDIEDVRR

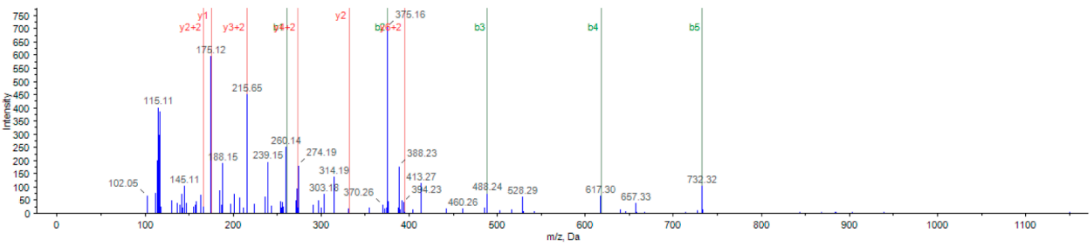

MS/MS for HbCPK9 peptied: INYEEFVAMMR

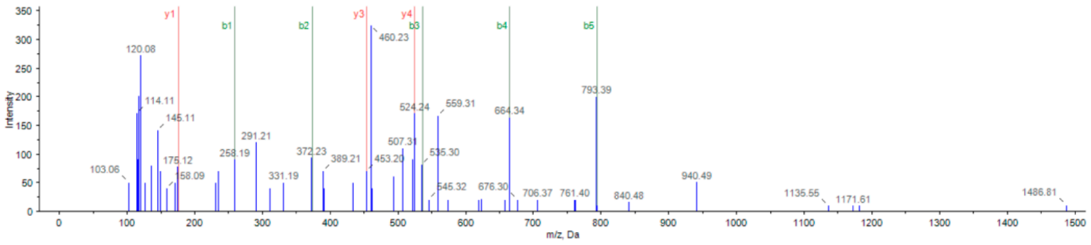

MS/MS for HbCPK9 peptied: LSAVEVLNHPWMR

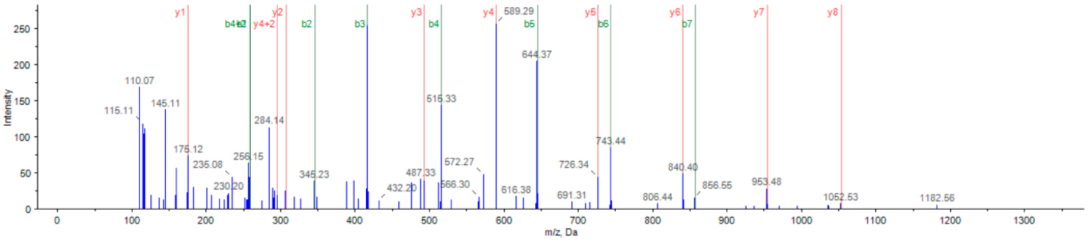

MS/MS for HbCPK9 peptied: LSESEVR

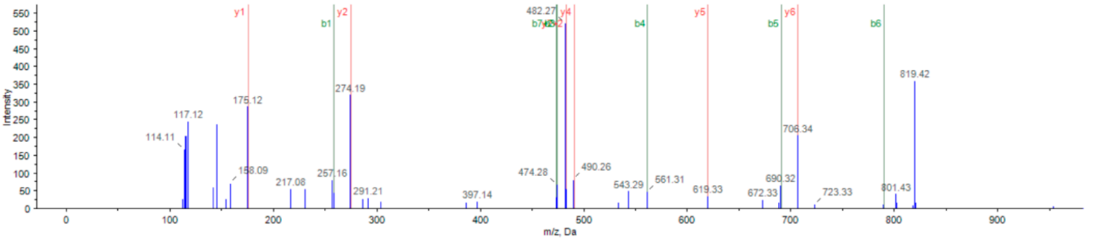

MS/MS for HbCPK9 peptied: NTYTFGR

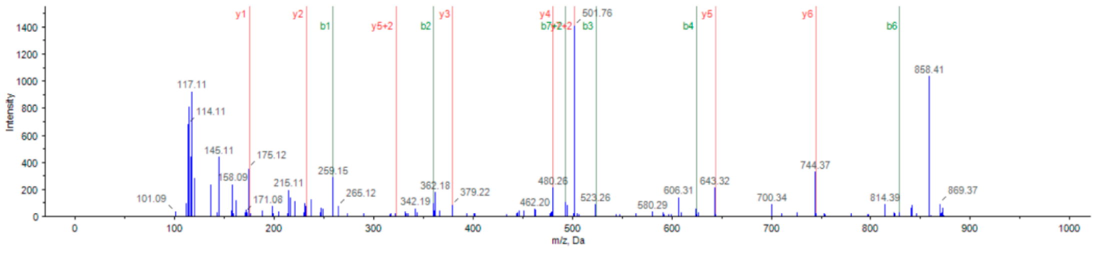

MS/MS for HbCPK9 peptied: SGYITMEELEHALK

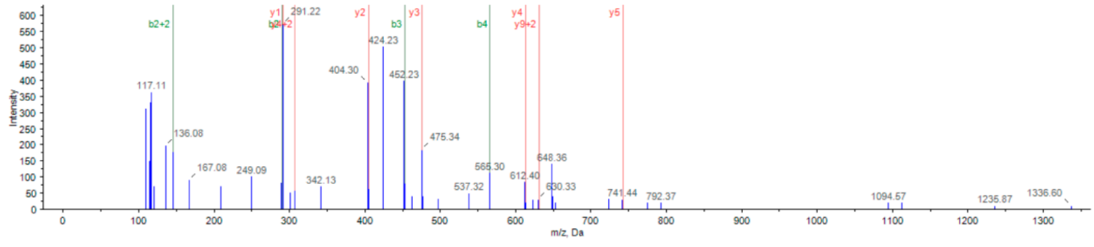

MS/MS for HbCPK9 peptied: DLVGSAYYVAPEVLHR

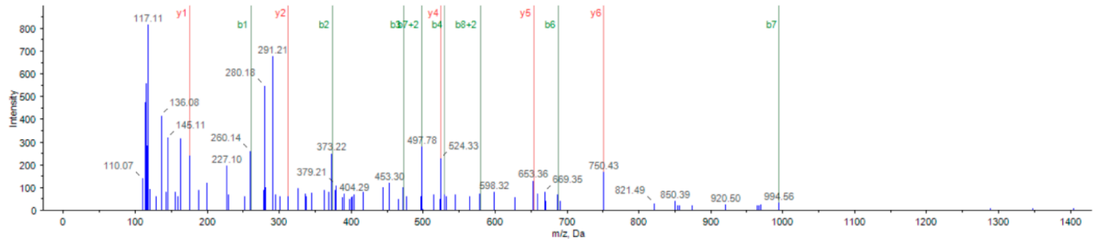

MS/MS for HbCPK9 peptied: SMDTDNSGTITYEELK

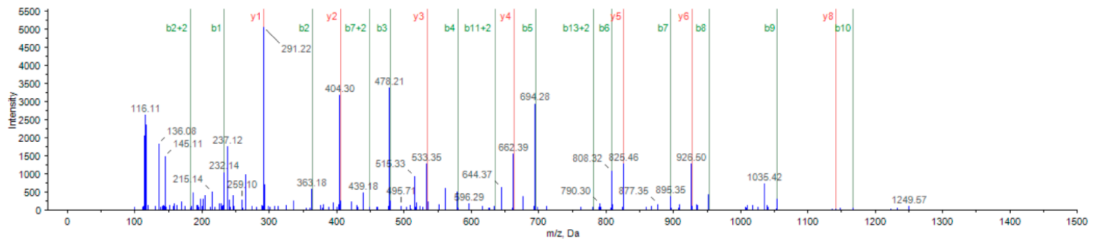

MS/MS for HbCPK9 peptied: VIAENLSEEEIMGLK

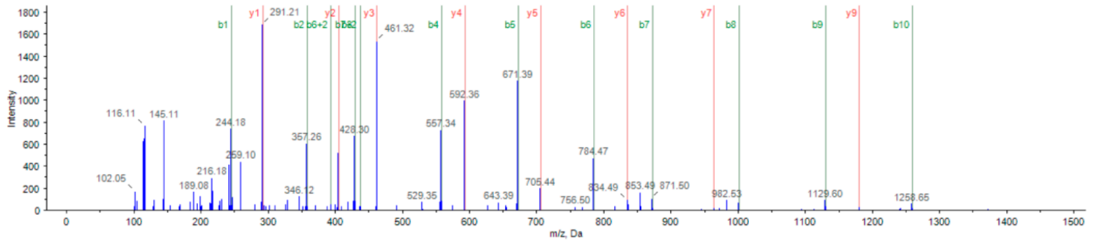

MS/MS for HbCPK9 peptied: GQFGVTYLVTHK

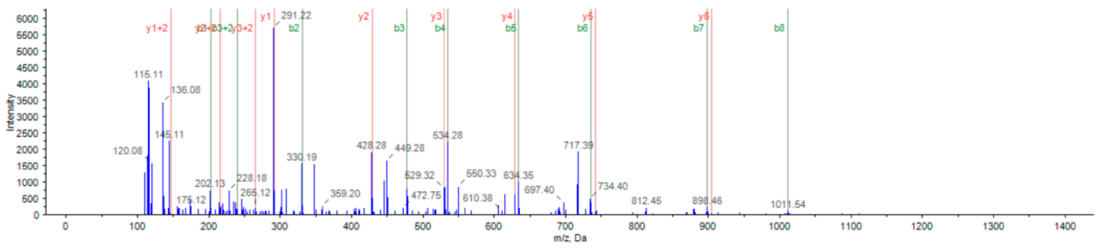

MS/MS for HbCPK9 peptied: EIIAEVDTDNDGR

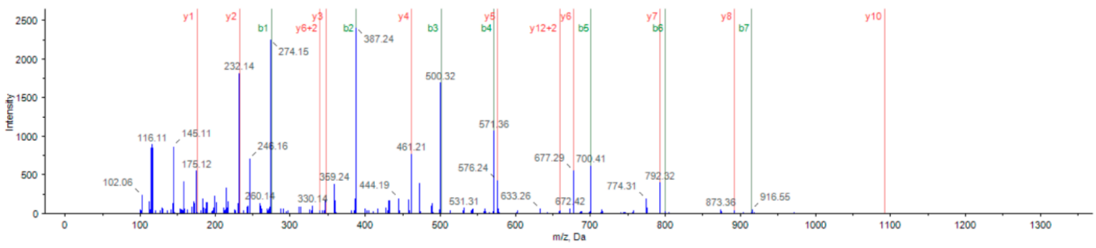

MS/MS for HbCPK9 peptied: EDGDASDKPLDIAVLTR

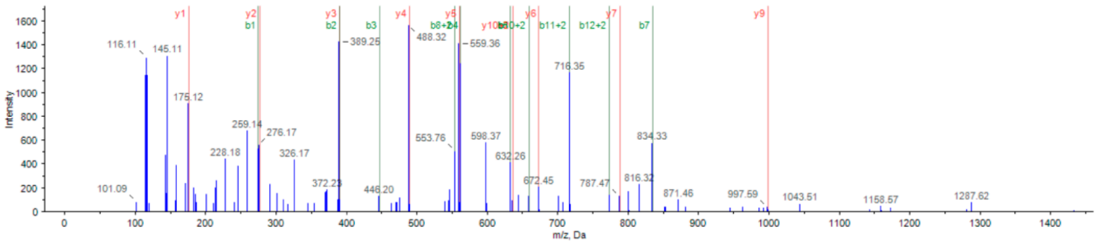

MS/MS for HbCPK9 peptied: DLKPENFLFLSTDENSPLK

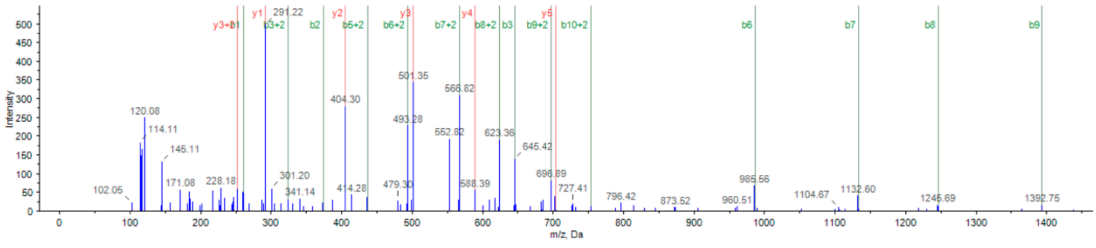

B

MS/MS spectrum mapped to HbCPK12

MS/MS for HbCPK12 peptied: VGSELMESEIK

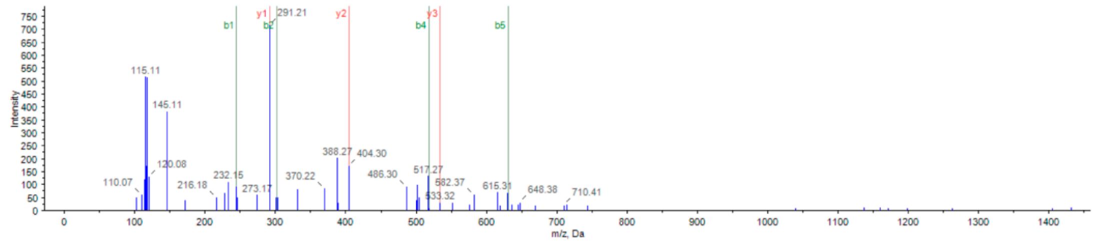

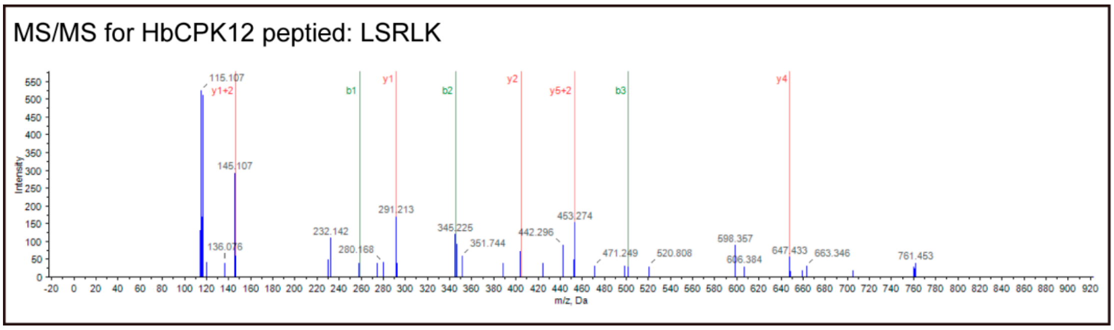

C

MS/MS spectrum mapped to HbCPK16

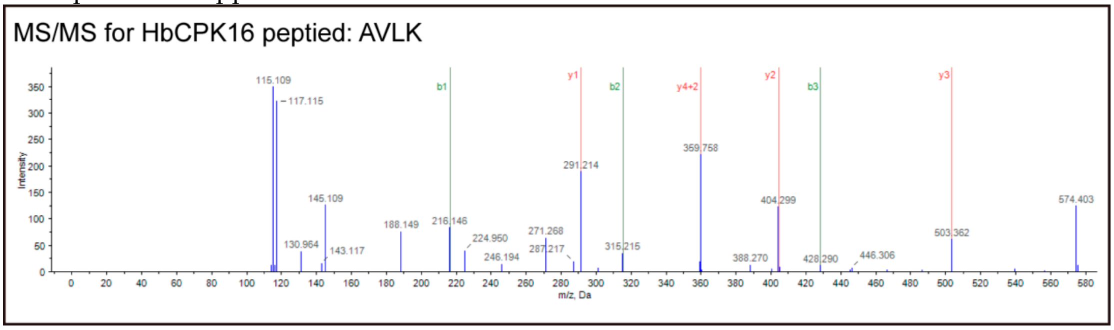

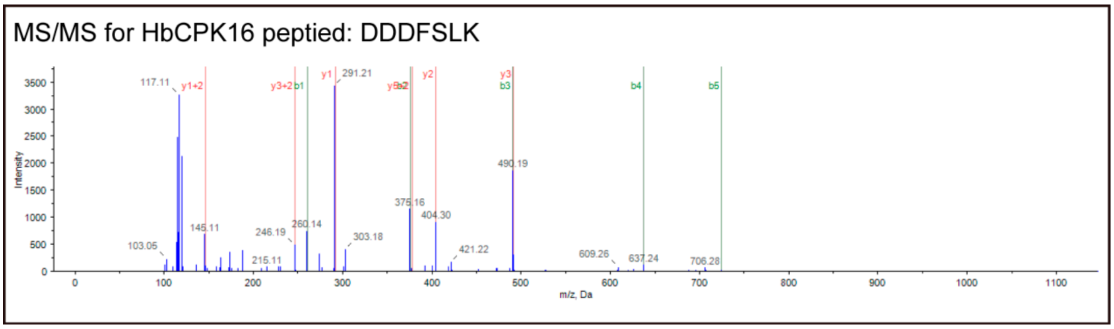

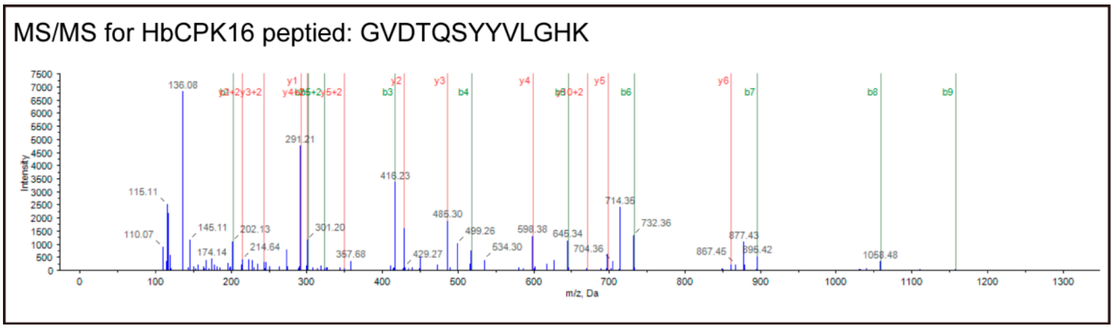

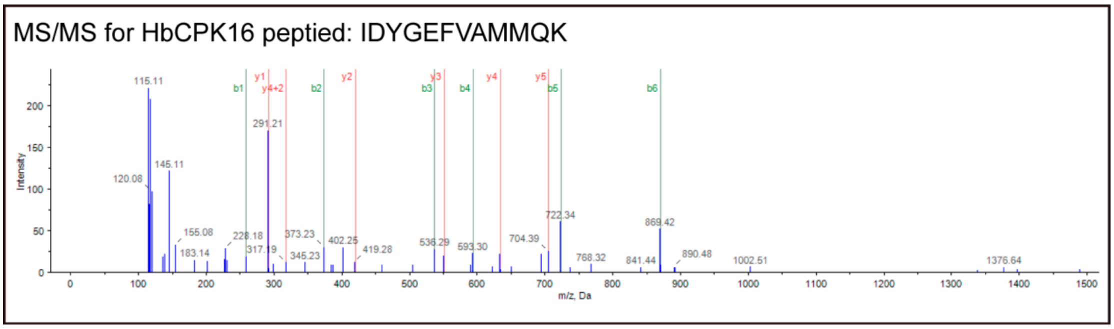

MS/MS for HbCPK16 peptied: LYQGYTQPEEQSTASHTKR

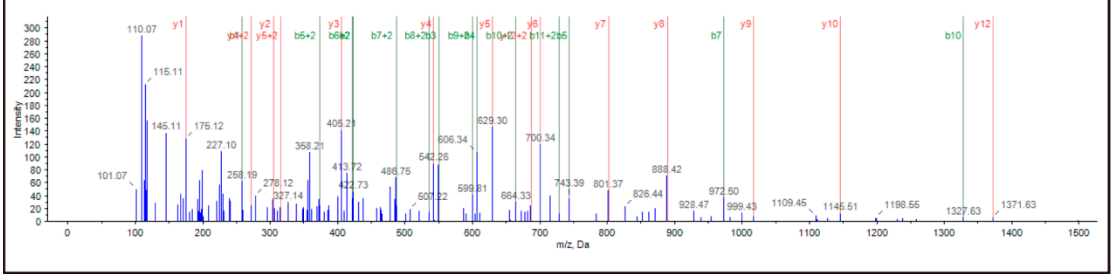

MS/MS for HbCPK16 peptied: VIAESLSEEEIAGLR

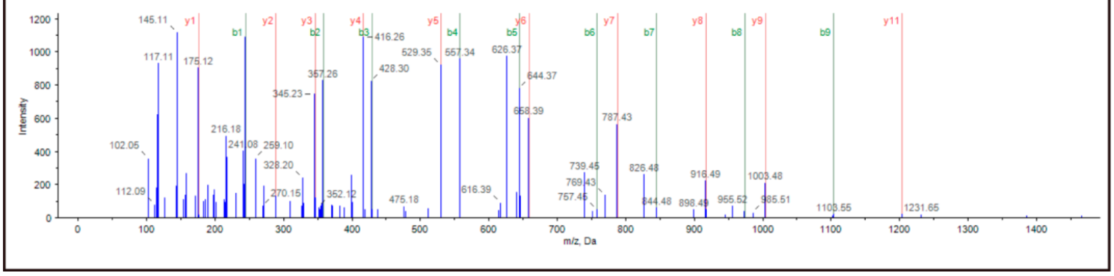

D

MS/MS spectrum mapped to HbCPK17

MS/MS for HbCPK17 peptied: ALMEAADIDK

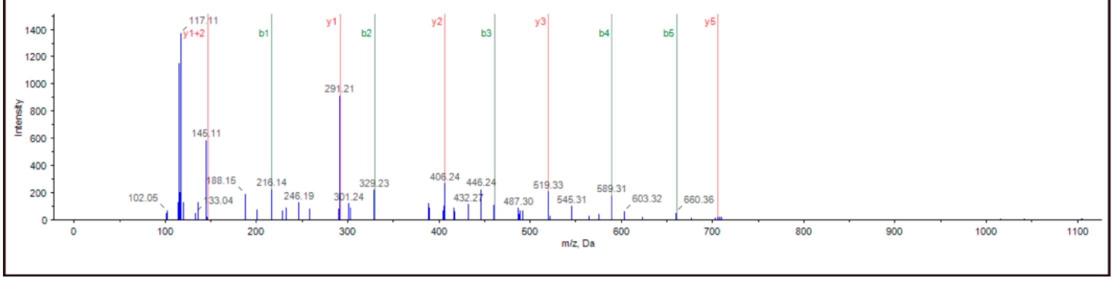

MS/MS for HbCPK17 peptied: DFGLDDVQLDEMIK

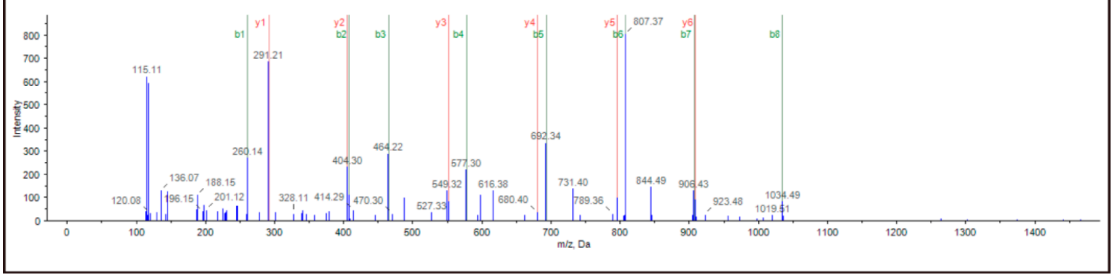

MS/MS for HbCPK17 peptied: DGSGYITVDELQQACK

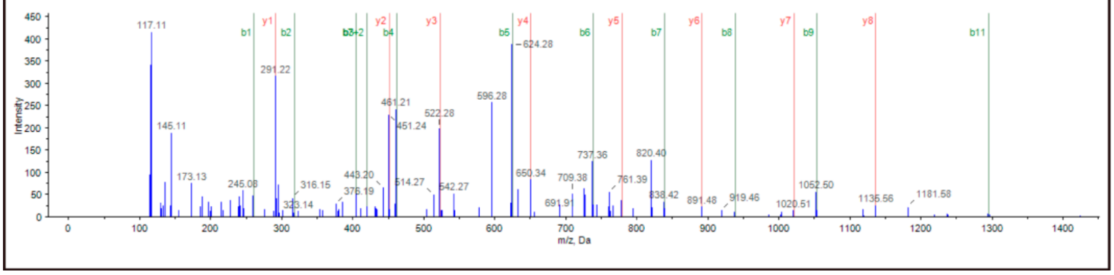

MS/MS for HbCPK17 peptied: IGQGQFGTTYLCTNK

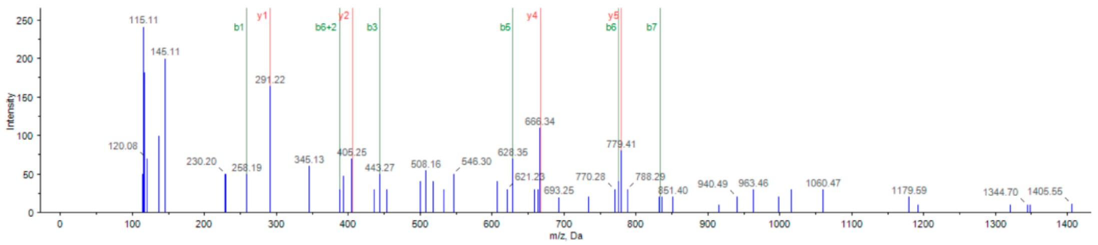

MS/MS for HbCPK17 peptied: ISAHEVLCNPWIVDDR

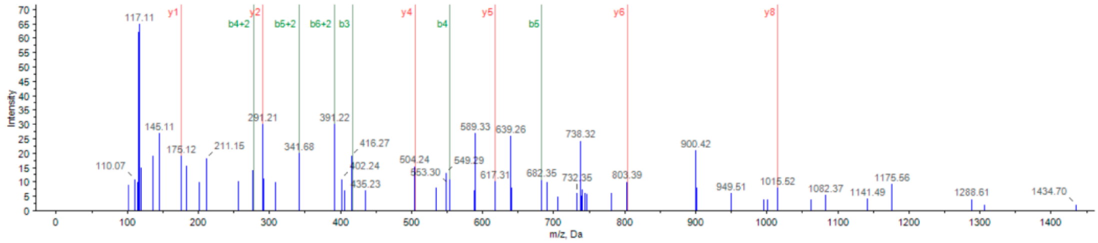

MS/MS for HbCPK17 peptied: LSEEEIGGLK

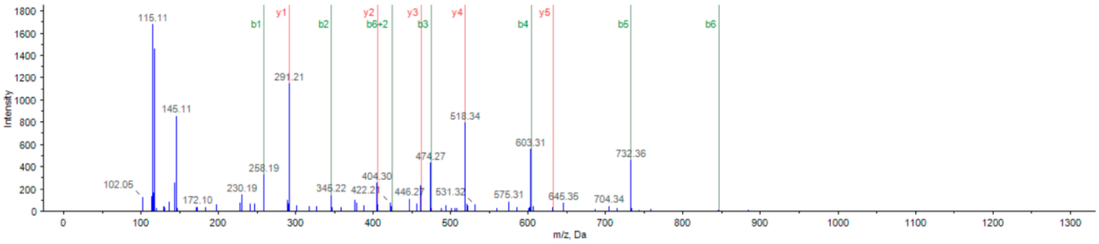

MS/MS for HbCPK17 peptied: MALHVIAER

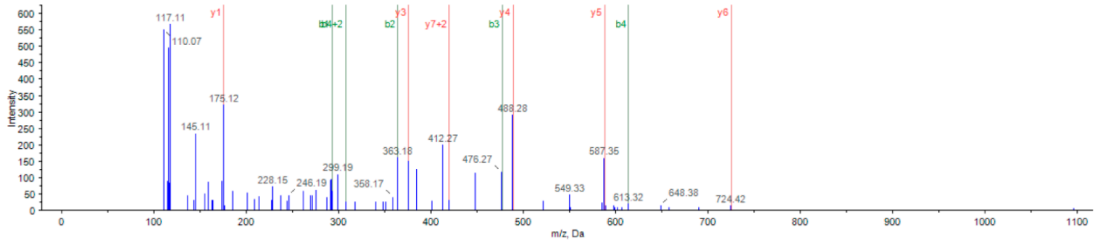

MS/MS for HbCPK17 peptied: TIVGVVEACHSLGVMHR

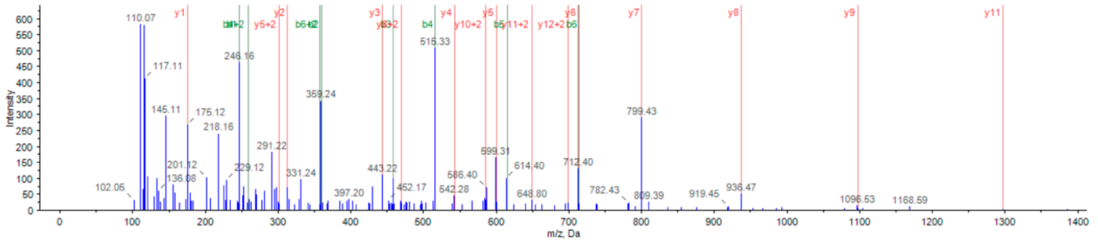

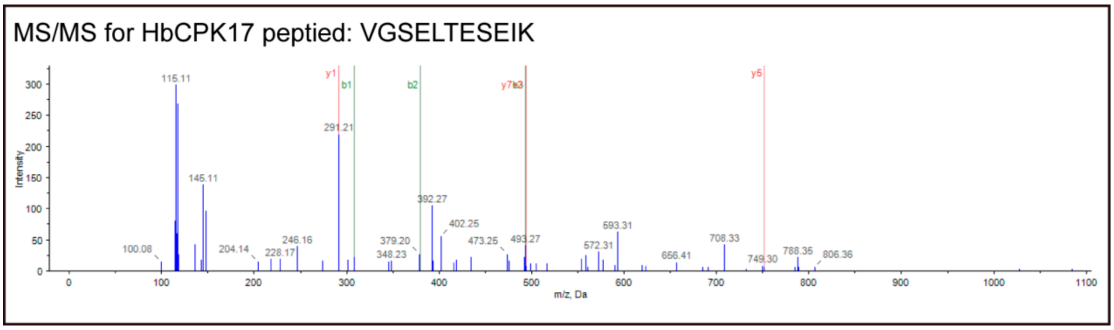

E

MS/MS spectrum mapped to HbCPK18

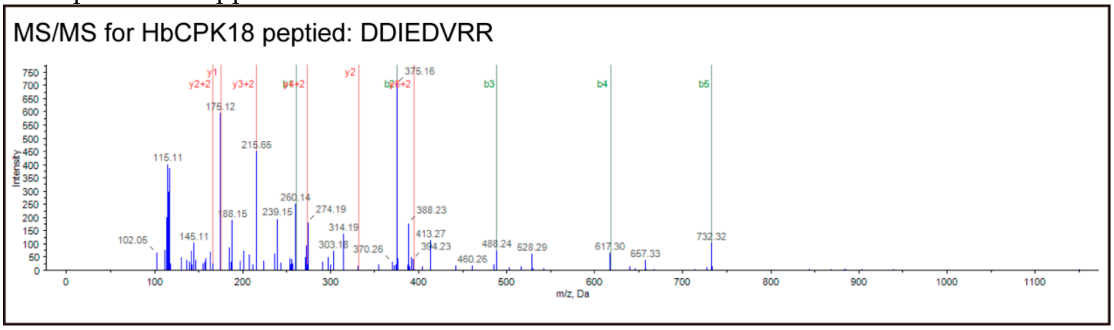

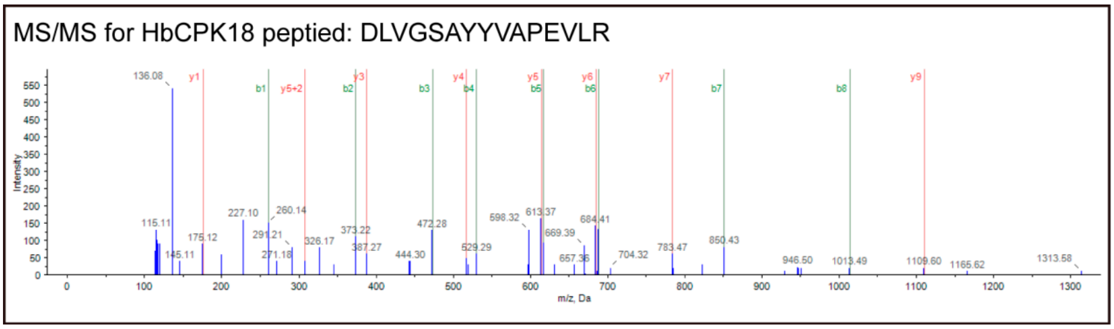

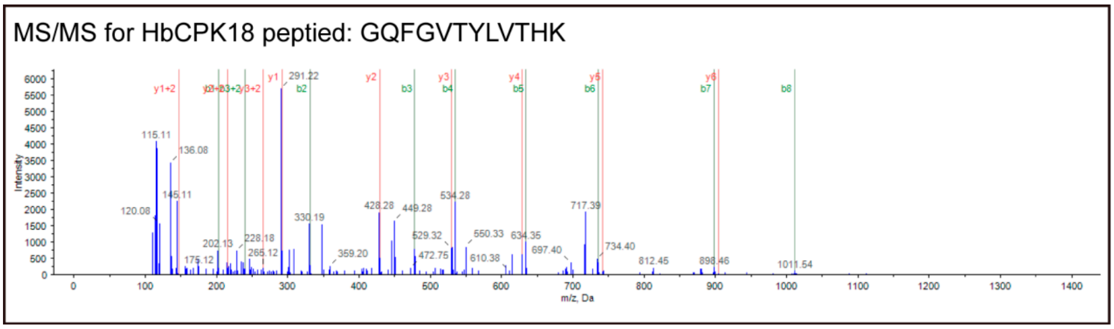

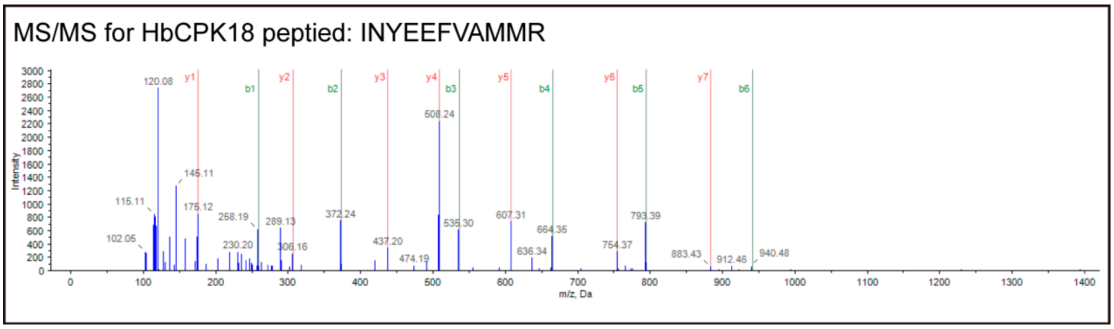

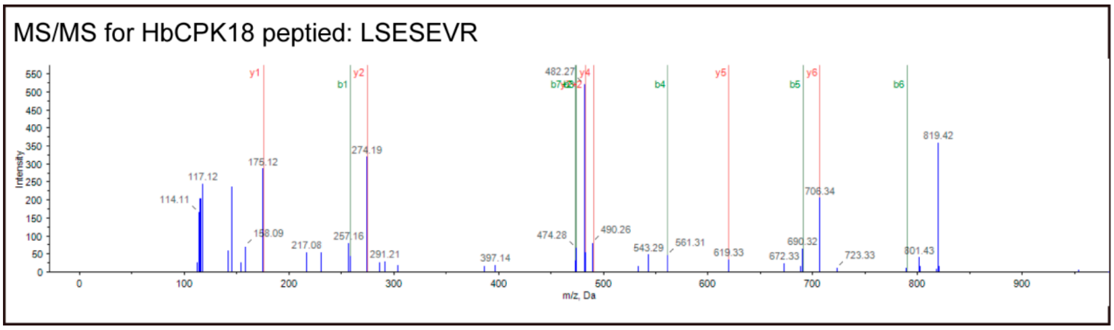

MS/MS for HbCPK18 peptied: LSESEVR

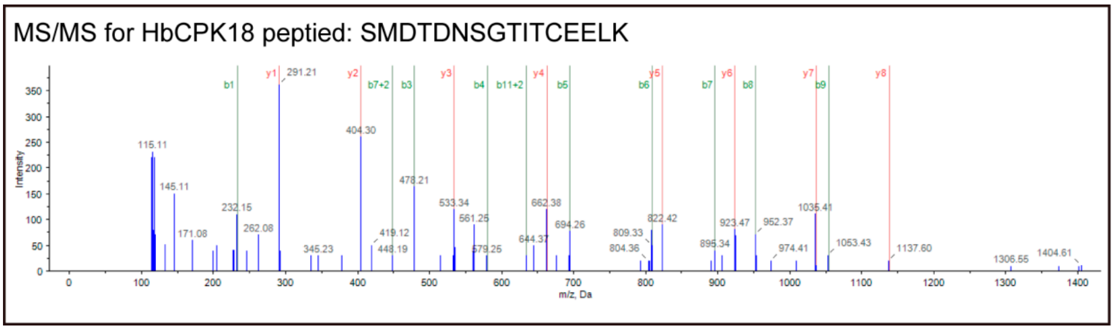

MS/MS for HbCPK18 peptied: SMDTDNSGTITCEELK

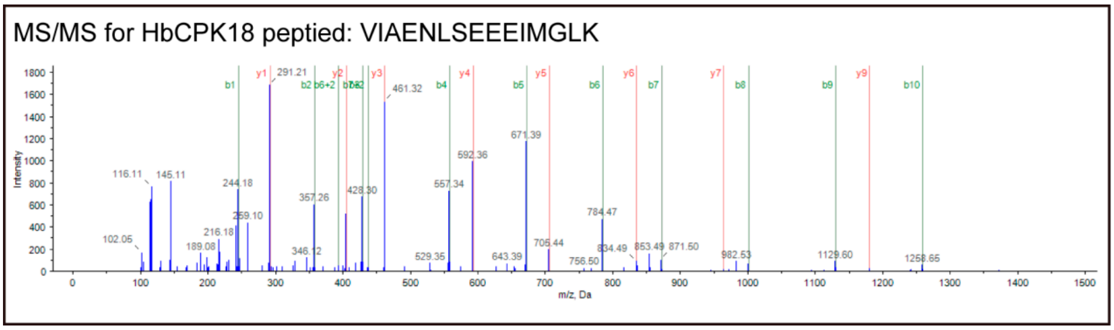

MS/MS for HbCPK18 peptied: VIAENLSEEEIMGLK

F

MS/MS spectrum mapped to HbCPK24

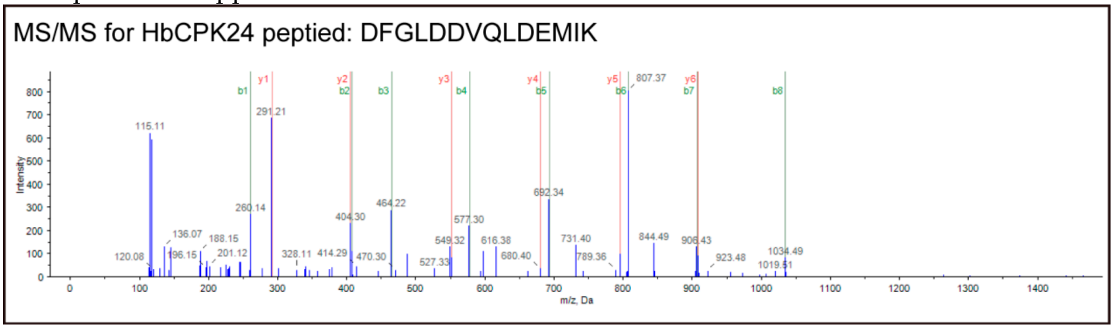

MS/MS for HbCPK24 peptied: DFGLDDVQLDEMIK

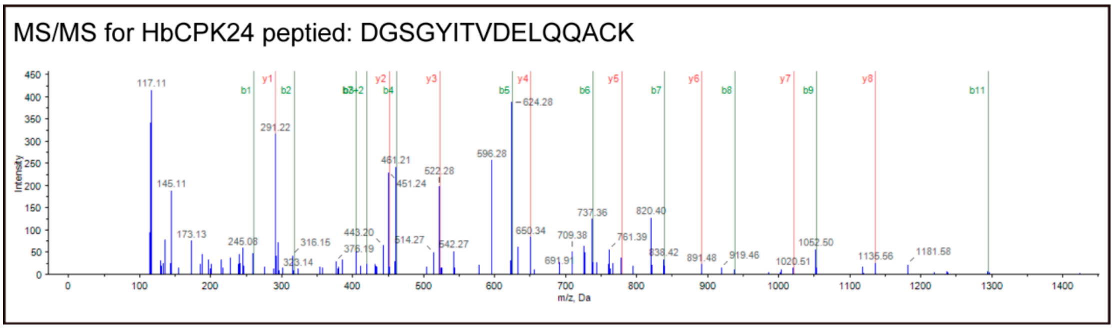

MS/MS for HbCPK24 peptied: DGSGYITVDELQQACK

MS/MS for HbCPK24 peptied: IDYSEFTTMMR

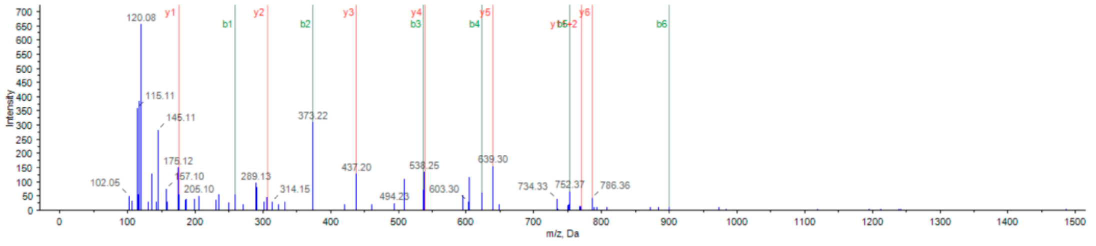

MS/MS for HbCPK24 peptied: ISAHEVLCNPWIVDDR

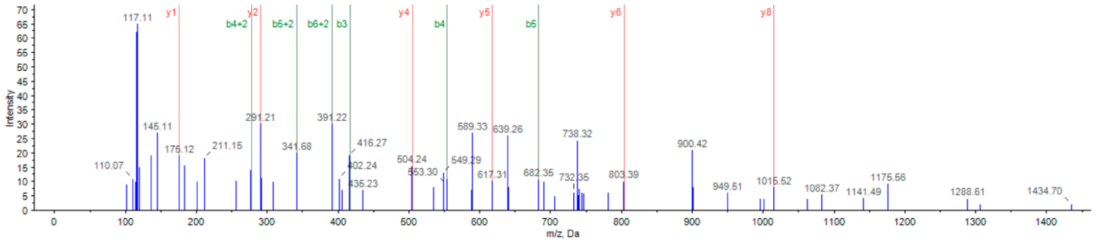

MS/MS for HbCPK24 peptied: LSEEEIGGLK

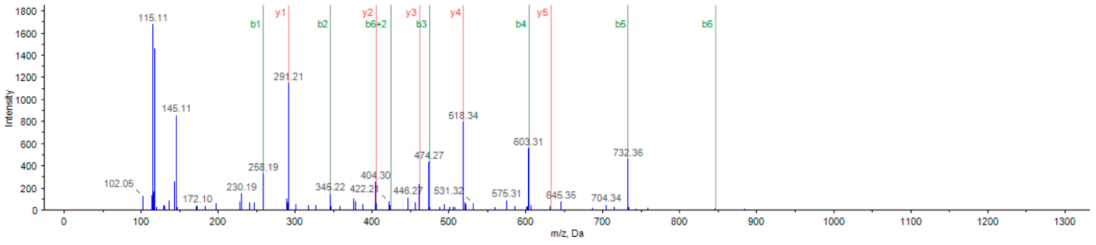

MS/MS for HbCPK24 peptied: QATSSSSSTTKPANTVLPYQTSR

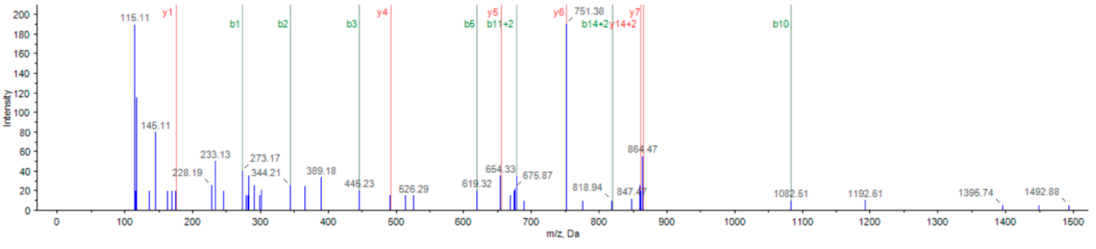

MS/MS for HbCPK24 peptied: VGSQLMESEIK

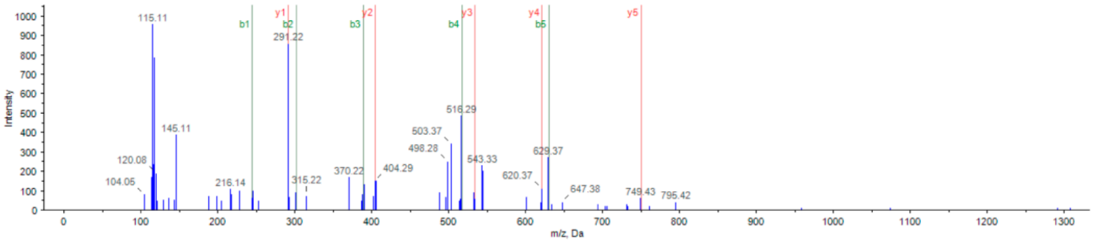

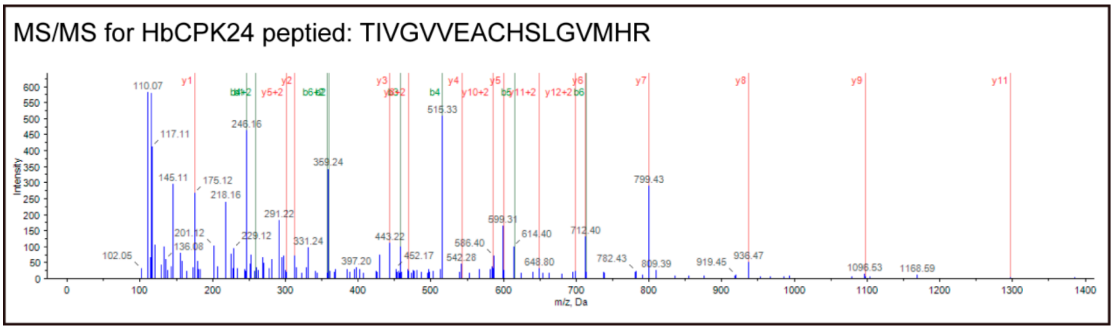

G

MS/MS spectrum mapped to HbCPK28

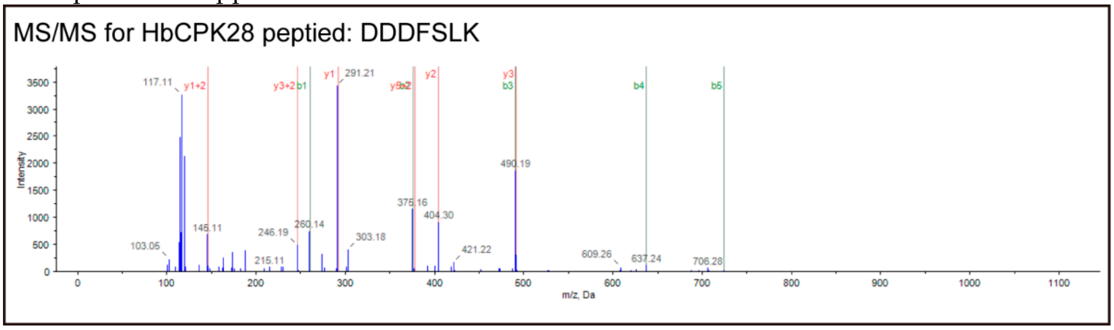

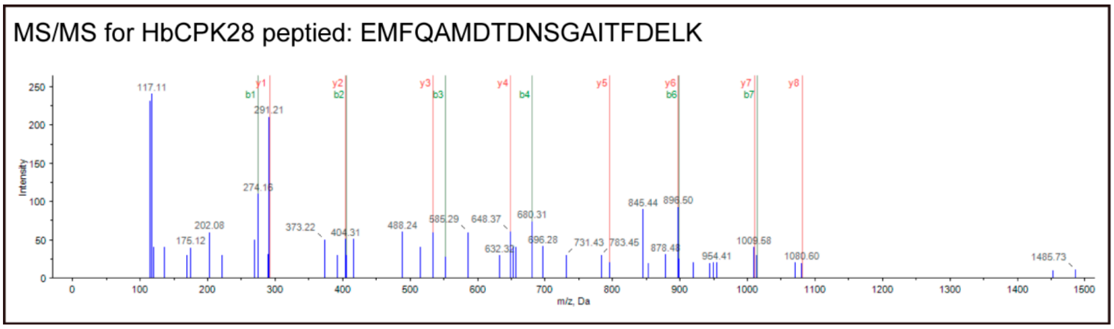

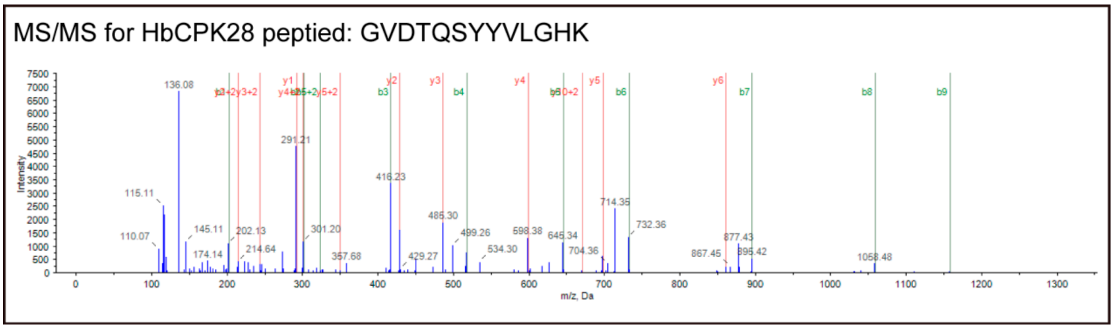

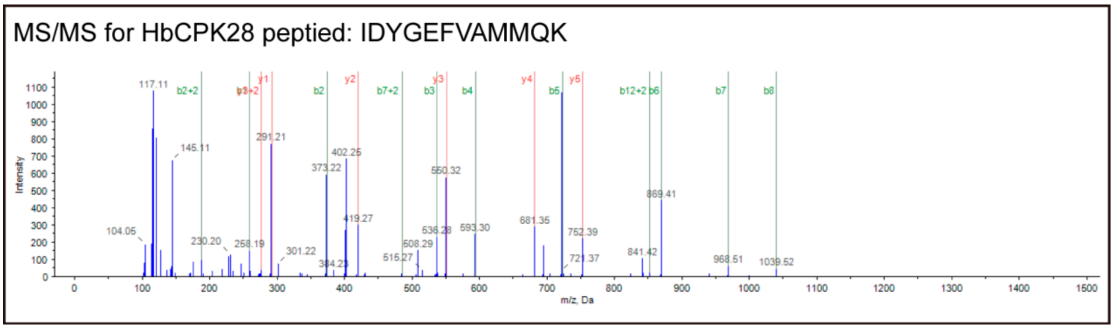

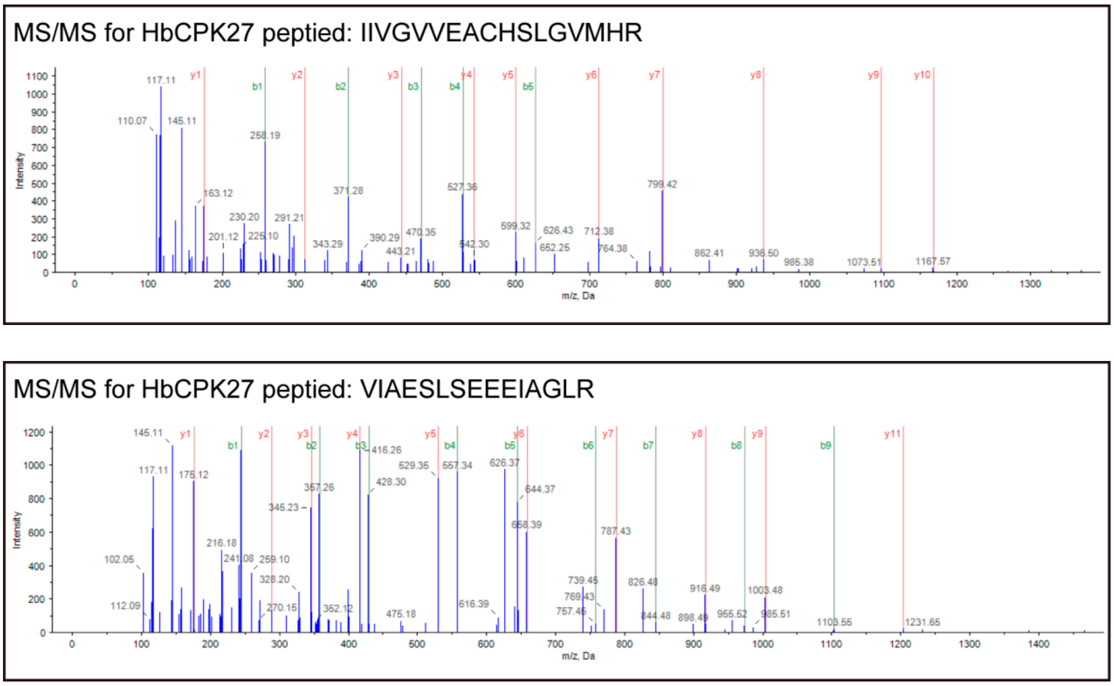

Figure S5. Raw spectra of peptides mapped to HbCPK proteins. The raw MS/MS spectra mapped to HbCPK proteins listed in Figure 1 were shown. The m/z values were indicated. The amino acids of identified peptides were also indicated at the top of each spectrum.
