# Supplementary material for: Calcium-Dependent Protein Kinase Family Genes Involved in Ethylene-Induced Natural Rubber Production in Different Hevea brasiliensis Cultivars
Source: Int J Mol Sci. 2018 Mar 22;19(4):947. doi: 10.3390/ijms19040947 (PMC5979512; doi:10.3390/ijms19040947)
Supplement: Supplementary file 1 [file ijms-19-00947-s001.zip › Supplementary files/Table S1.pdf]

Table S1. Detailed information of *HbCPKs*.

		Predicted Protein[b]			
**Gene Name**	**Scaffold Location** [a]	**ORF (bp)**	**Length (aa)**	**MW (kDa)**	***p*I**
HbCPK1	scaffold0043 (+): 2123951 - 2130764	1767	589	65.75	5.22
HbCPK2	scaffold0056 (+): 1057685 - 1060286	1605	535	59.45	5.40
HbCPK3	scaffold0073 (−): 399919 - 406748	1653	550	62.47	6.00
HbCPK4	scaffold0152 (+): 1897945 - 1904245	1605	535	60.13	6.75
HbCPK5	scaffold0153 (−): 1475372 - 1481763	1629	543	61.34	5.86
HbCPK6	scaffold0184 (−): 1457576 - 1465083	1725	575	63.87	5.14
HbCPK7	scaffold0233 (−): 1368833 - 1371794	1572	524	58.21	5.47
HbCPK8	scaffold0277 (−): 290091 - 295708	1650	550	62.65	6.73
HbCPK9	scaffold0280 (+): 1180129 - 1185851	1593	531	59.63	6.13
HbCPK10	scaffold0349 (−): 97495 - 104134	1308	436	49.19	5.17
HbCPK11	scaffold0354 (−): 908601 - 912058	1605	535	60.20	6.07
HbCPK12	scaffold0389 (+): 807679 - 813991	1503	501	56.60	5.54
HbCPK13	scaffold0570 (−): 766045 - 771889	1596	532	60.38	6.25
HbCPK14	scaffold0587 (+): 458096 - 462475	1587	529	59.95	6.11
HbCPK15	scaffold0591 (+): 129182 - 134548	1671	556	62.78	6.20
HbCPK16	scaffold0592 (−): 155885 - 163397	1686	562	62.87	5.54
HbCPK17	scaffold0704 (−): 560203 - 565100	1554	518	58.17	5.36
HbCPK18	scaffold0770 (−): 364598 - 371552	1593	531	59.57	6.09
HbCPK19	scaffold0846 (+): 3570 - 14765	1584	528	59.43	5.91
HbCPK20	scaffold0924 (−): 29764 - 47109	1584	528	59.37	5.86
HbCPK21	scaffold0972 (+): 89336 - 95624	1698	566	63.98	8.97
HbCPK22	scaffold1079 (−): 93442 - 105046	1698	566	64.07	9.25
HbCPK23	scaffold1156 (−): 177750 - 181402	1656	552	62.62	5.68
HbCPK24	scaffold1358 (−): 115651 - 120562	1509	503	56.75	5.38
HbCPK25	scaffold1505 (+): 82032 - 85759	1755	585	65.65	5.44
HbCPK26	scaffold1583 (+): 10113 - 22975	1773	590	65.79	5.23
HbCPK27	scaffold1681 (+): 601 - 5049	1602	534	60.57	5.98
HbCPK28	scaffold2028 (+): 43 - 9335	1686	562	62.80	5.62
HbCPK29	scaffold2327 (+): 14881 - 18420	1896	632	70.79	5.47
HbCPK30	scaffold4186 (−): 855 - 5120	1605	535	60.63	6.49

a. scaffold location: [Scaffold number (Orientation): start - end], "+" and "−" indicated the forward and reverse orientation, respectively; b. aa, amino acid; MW, molecular weight; *p*I, isoelectric point; c. The predicted subcellular location of each HbCPK was analyzed using Softberry server.
