# Supplementary material for: Calcium-Dependent Protein Kinase Family Genes Involved in Ethylene-Induced Natural Rubber Production in Different Hevea brasiliensis Cultivars
Source: Int J Mol Sci. 2018 Mar 22;19(4):947. doi: 10.3390/ijms19040947 (PMC5979512; doi:10.3390/ijms19040947)
Supplement: Supplementary file 1 [file ijms-19-00947-s001.zip › Supplementary files/Table S2.pdf]

Table S2. Primers used in this work.

Primer Name		Primer Sequence (5'-3')	Production Size (bp)
HbCPK1	sense	CTTGGGCGATATTCAACTGGC	121
	anti-sense	CCGGTCTGACCAGCGACA	
HbCPK2	sense	GGCACTATTACACTTGAGGAGCTG-	268
	anti-sense	GCATTCCAAATTCGCGGAGT	
HbCPK3	sense	GAAGTGGCTGATGTTGACGGA	203
	anti-sense	AGCACATCATTATCAGTTTCACCA	
HbCPK4	sense	GCTGATGTTGATGGTAACGGGA	133
	anti-sense	TGAACCCACTTTTGTCCTTGTC	
HbCPK5	sense	ATGGGTTCTTCCCTACCGGACT	233
	anti-sense	TCCTCACCACATTAGGGTGCT	
HbCPK6	sense	TGCCATTCATTGGGGGTG	200
	anti-sense	GCTTCGGGGCCATATTGTT	
HbCPK7	sense	CCACGCAAGGGACAAAGCTAT	231
	anti-sense	GCATTCCGAAATCGCGAAG	
HbCPK8	sense	GGCTGATGTTGATGGGAATG	223
	anti-sense	GACAACTTCACGCATGATGTCTT	
HbCPK9	sense	CGAACAGGCTATATTTGATTCTGT	131
	anti-sense	AAAGTCGGTCCTTAGGATCAGTT	
HbCPK10	sense	GCTACGACTTACAGCTCAGCAAGT	172
	anti-sense	CTCCACCGACAAATGCTCAG	
HbCPK11	sense	GCAGGAGGCGAGTTGTTTGA	192
	anti-sense	ATCAGTTGCCTTCAAAAGGG	
HbCPK12	sense	GTGTCAGCTTTCTCGTTCTTTGAC	248
	anti-sense	GTCAATCCTAAAGCATCTCCCAAG	
HbCPK13	sense	CAGATGTGCCACAAGCATGG	233
	anti-sense	AGTATGACACCAGCACTCCAGAC	
HbCPK14	sense	ATTTCTCCTGCGAATCCACC	113
	anti-sense	ATCCCAAACTGACCTCTACCC	
HbCPK15	sense	TGCTAAAGGGCATTATTCGG	105
	anti-sense	TTGAGATCACGGTGCATCACTC	
HbCPK16	sense	TAGACGGACTATGAGAAACAGTCTG	211
	anti-sense	TCTTTTCAAGCCTCTAATGTGCC	
HbCPK17	sense	CCAACCGCTAGCACTAACTGATTC	230
	anti-sense	CGGCATAGACGAATTCTAACTGTC	
HbCPK18	sense	GATCCTAAGGCCCGACTTTC	284
	anti-sense	TTGGTACCCAATTTTGGAATACTA	
HbCPK19	sense	GGTGATGTTGTCAAGTCAAGACTAA	141
	anti-sense	ATCAGTATCCATCTTCCTGAAC	
HbCPK20	sense	GGACTTCGTAATTTTAACTCCCAGC	223
	anti-sense	TCAATGCATCCCGGAGTTCAT	
HbCPK21	sense	GGACTTGTTGATTTCTCCGAAT	295
	anti-sense	CAGATGTGCTTGGTGCATTTC	
HbCPK22	sense	GGACTCCCGTGTCCTGGAGATT	237
	anti-sense	AGTGGGTCAATGGAGCCTTTTA	
HbCPK23	sense	TCGCCAAGGTCGACGAGTATC	225
	anti-sense	CGATATAGTCTTGCACGCAAAGG	
HbCPK24	sense	TCAGGACAACGATGGACGAA	74
	anti-sense	TTCCAAATTCTTCATCGCCC	
HbCPK25	sense	CCGCCTCTAGAACCTGAGGATAT	128
	anti-sense	GCTGGTCTACTCTCTTCTCTGCG	

HbCPK26	sense	TCTGAGGGTTCTATGAACGGATC	191
	anti-sense	TTATTGGGTTTCTTCGGCTTCT	
HbCPK27	sense	ACGCCGAGGGCGACAAGTAT	196
	anti-sense	CTATTTCTGTCCTCAGCTTCGCC	
HbCPK28	sense	GACGAACTATGCGAAACAGTCTG	150
	anti-sense	GAGCAAGGAAGCAAAATCGC	
HbCPK29	sense	AATGATGGTCGGATAGACTACAATG	133
	anti-sense	ATCAACAAACTTGTAGTGCCTCC	
HbCPK30	sense	GCTAAGAGATGCGTTGGCTGA	238
	anti-sense	CTACCCCCCGTTGTTCATCTG	
HbActin	sense	CAGGGCAGTGTTTCCCAGTATAG	379
	anti-sense	CAGCACGATACCAGTTGTACGAC	
