# Supplementary material for: Calcium-Dependent Protein Kinase Family Genes Involved in Ethylene-Induced Natural Rubber Production in Different Hevea brasiliensis Cultivars
Source: Int J Mol Sci. 2018 Mar 22;19(4):947. doi: 10.3390/ijms19040947 (PMC5979512; doi:10.3390/ijms19040947)
Supplement: Supplementary file 1 [file ijms-19-00947-s001.zip › Supplementary files/Table S3.pdf]

Table S3. Putative *cis*-element sequences in promoter regions of *HbCPK* genes.

Classification	Symbol	*cis*-Element	Sequence *	Description
Transcription initiation	T	TATA box	TATAAAT	Core *cis*-element influenced the efficiency of transcription in eucaryote
	C	CAAT box	CCAAT	Common *cis*-element in eukaryotic promoter and enhancer regions
Phytohormone responsiveness	A	ABRE	TACGTG	Abscisic acid (ABA)-responsive element
	E	ERE	AWTTCAAA	Ethylene-responsive element
	G	GARE	WAACAR	Gibberellin-responsive element
	P	P-box	CCTTTTG	Gibberellin-responsive element
Stress responsiveness	M	MBS	YAACTG	MYB binding site involved in drought-inducibility
	K	MRE	AACCTAA	MYB binding site involved in light responsiveness
	H	HSE	CTNGAANNTTCNAG	Heat shock-responsive element
	D	TC-rich	RTTTTCTYMM	*cis*-element involved in defense and stress responsiveness
	J	G-box	YACGTN	*cis*-regulatory element involved in light responsiveness
	I	I-box	GATAWGR	Part of a light responsive element
	P	ARE	TGGTTT	*cis*-acting regulatory element essential for the anaerobic induction
	F	AE-box	AGAAACTT	part of a module for light response
	W	WUN-motif	TCATTACGAA	Wound-responsive element
High transcription	U	5'UTR Py-rich stretch	TTTCTTCTCT	*cis*-regulatory element conferring high transcription levels

*, N = A, C, G or T; M = A or C; R = A or G; W = A or T; Y = C or T.
